# Fibrinogen Mitigates Prion-Mediated Platelet Activation and Neuronal Cell Toxicity

**DOI:** 10.3389/fcell.2022.834016

**Published:** 2022-03-21

**Authors:** Deepa Gautam, Jyotsna Kailashiya, Arundhati Tiwari, Rameshwar Nath Chaurasia, Gowtham K. Annarapu, Prasenjit Guchhait, Debabrata Dash

**Affiliations:** ^1^ Center for Advanced Research on Platelet Signaling and Thrombosis Biology, Department of Biochemistry, Institute of Medical Sciences, Banaras Hindu University, Varanasi, India; ^2^ Department of Neurology, Institute of Medical Sciences, Banaras Hindu University, Varanasi, India; ^3^ Regional Centre for Biotechnology, National Capital Region Biotech Science Cluster, Faridabad, India

**Keywords:** platelet-derived extracellular vesicles, calpain activity, intracellular calcium, neuronal cell toxicity, mitochondrial membrane potential, fibrinolysis

## Abstract

Prion peptide (PrP) misfolds to infectious scrapie isoform, the β pleat-rich insoluble fibrils responsible for neurodegeneration and fatal conformational diseases in humans. The amino acid sequence 106–126 from prion proteins, PrP(106–126), is highly amyloidogenic and implicated in prion-induced pathologies. Here, we report a novel interaction between PrP(106–126) and the thrombogenic plasma protein fibrinogen that can lead to mitigation of prion-mediated pro-thrombotic responses in human platelets as well as significant decline in neuronal toxicity. Thus, prior exposure to fibrinogen-restrained PrP-induced rise in cytosolic calcium, calpain activation, and shedding of extracellular vesicles in platelets while it, too, averted cytotoxicity of neuronal cells triggered by prion peptide. Interestingly, PrP was found to accelerate fibrin-rich clot formation, which was resistant to plasmin-mediated fibrinolysis, consistent with enhanced thrombus stability provoked by PrP. We propose that PrP-fibrinogen interaction can be clinically exploited further for prevention and management of infectious prion related disorders. Small molecules or peptides mimicking PrP-binding sites on fibrinogen can potentially mitigate PrP-induced cellular toxicity while also preventing the negative impact of PrP on fibrin clot formation and lysis.

## Introduction

The cellular prion protein (PrP^c^) is approximately 33–35 kDa, a glycosyl-phosphatidylinositol (GPI)-anchored protein being expressed on different cell types and tissues (Chiesa, 2015). It is fundamentally an α-helical structure that gets metamorphosed by unknown mechanisms into infectious scrapie isoform (PrP^Sc^), which is primarily composed of β-pleated insoluble fibrils (Aguzzi and Calella, 2009). The latter form aggregates in extracellular matrix causing fatal prion-related diseases such as transmissible spongiform encephalopathies (TSEs) which include Creutzfeldt–Jakob disease (CJD) in humans, scrapie in sheep and goat as well as bovine spongiform encephalopathy (BSE) in cattle (Aguzzi and Calella, 2009). Exposure of neuronal cells to prion peptide elicits synthesis of proteinase K-resistant transmembrane prion protein, which is known to mediate neurotoxicity (Fang et al., 2016).

Amyloid and prion are responsible for a spectrum of conformational pathologies, including Alzheimer’s disease, Parkinson’s disease, amyloidosis, and TSEs in humans (Sabate, 2014). The basic defect in such diseases is abnormal conformation of proteins due to misfolding, resulting in a fibrillar structure that gets deposited in tissues and leads to toxicity (Sabate, 2014). Shreds of evidence even show that prions can assemble as amyloid, which can then convert into prions (Watts and Prusiner, 2018). Prions are highly infectious oligomeric proteins that can infect through oral and parenteral routes (Geschwind, 2015). The prion peptide is pathogenically transferred to the central as well as peripheral nervous system that finally leads to development of prion disease in humans. These proteins can be transferred through circulation, further infecting and converting other proteins to the PrP^Sc^ form (Aguzzi and Calella, 2009; Caughey et al., 2009; Geschwind, 2015).

Platelets, circulating enucleate cells procured from megakaryocytes, are responsible for hemostasis. Hyperactivity of platelets, however, can lead to ischemic thrombotic disorders such as acute myocardial infarction and cerebral stroke, the two major causes of mortality worldwide (Previtali et al., 2011). Expression of PrP^c^ mRNA and its cognate protein in platelets has been reported earlier (Starke et al., 2005). Resting platelets express PrP^c^ on intracellular membranes from where it is translocated to the cellular surface as well as released into microvesicles and exosomes upon cell activation (Starke et al., 2005). PrP^c^ participates in physiological functions including neurotransmission, neurodevelopment, and protection from toxins and oxidative damage, possibly due to its ability to interact with other proteins (Chiesa, 2015). We have earlier demonstrated that PrP(106–126) induces rise in platelet intracellular calcium, leading to upregulation of calpain activity and release of extracellular vesicles, the hallmarks of platelet activation (Mallick et al., 2015). There is evidence supportive of interaction between amyloid β peptide and plasma fibrinogen (Aguzzi and Calella, 2009; Chiesa, 2015) that can lead to abnormal clot formation and, thus, contribute to vascular pathology in Alzheimer’s disease (Aguzzi and Calella, 2009; Cortes-Canteli and Strickland, 2009; Cortes-Canteli et al., 2010; Zamolodchikov et al., 2016). We report attenuation in amyloid β-induced platelet activation and amyloid β accumulation in neuronal cells in the presence of fibrinogen (Fang et al., 2016; Sonkar et al., 2016). Because both amyloid β and infectious prion peptide share a β-sheet structure and can possibly convert to or mimic each other (Stohr et al., 2008; Makarava et al., 2011; Watts and Prusiner, 2018), we asked whether prion peptide, too, can interact with fibrinogen similar to amyloid β. In this study, we examine the interaction of PrP^Sc^ (106–126) with fibrinogen, and its effects on prion-mediated platelet activation responses as well as neuronal toxicity, dynamics of fibrin clot.

## Materials and Methods

PrP(106–126) (KTNMKHMAGAAAAGAVVGGLG) was purchased from Tocris Biosciences (#3491). It was dissolved in 1 ml buffer B (20 mM HEPES, pH 7.4, 138 mM NaCl, 2.9 mM KCl, 1 mM MgCl_2_, and 0.36 mM NaH_2_PO_4,_ supplemented with 5 mM glucose) at 0.5 mM stock concentration and aliquoted at −20°C. Human neuroblastoma cell line SH-SY5Y was purchased from National Center for Cell Sciences, Pune. DMEM/F12 (Dulbecco’s modified Eagle medium) (#CC3022) was procured from Cell clone, and heat-inactivated fetal bovine serum (#10082147), trypsin/EDTA solution (#25200072) and antibiotic and antimycotic solutions (100X) (#A002) were from Gibco. Calcium ionophore A23187 (#C7522), CCCP (#21855), 2′, 7′-dichlorodihydrofluorescein diacetate (H_2_DCFDA) (#D6883), EGTA (#E-4378), EDTA (#18454), sodium orthovanadate (#S6508), acetylsalicylic acid (#A5376), thrombin (#T6884), fibrinogen (#F4883), plasminogen (#P9156), tissue plasminogen activator (#T2943), dimethylsulfoxide (DMSO) (#D5879) and mouse monoclonal anti-talin antibody (#T3287) were purchased from Sigma. Fura 2-AM (#F-0888) was acquired from Calbiochem. *Glycine* and reagents for electrophoresis were from Merck. Polyvinylidene fluoride (PVDF) membranes and enhanced chemiluminescence detection kit were from Millipore. Flow cytometry sheath fluid was from BD Biosciences. Alexa fluor 488-fibrinogen (#F13191), t-Butoxycarbonyl-Leu-Metchloromethylcoumarin (calpain substrate) (#A6520), phalloidin-FITC (#F432), MitoTracker-Red (#M7512) were from Invitrogen, and horseradish peroxidase (HRP)-labeled anti-rabbit IgG was from Genei (Bangalore). Propidium iodide (#11195) was obtained from SRL. All other reagents were of analytical grade. Type I deionized water (18.2 MΩ⋅cm, Millipore) is used in all experiments.

### Human Washed Platelet Preparation

Human blood was collected from antecubital veins of healthy volunteers in citrated-phosphate-dextrose-adenine under informed written consent, strictly as per the recommendations and approval of the Institutional Ethical Committee of Banaras Hindu University. The study methodologies conformed to the standards set by the Declaration of Helsinki. Platelets were isolated by differential centrifugation as reported earlier (Gautam et al., 2019). Briefly, blood was centrifuged at 200 g for 10 min. Platelet-rich plasma (PRP) was collected carefully to avoid the contamination of red and white blood cells and was incubated with 1 mM acetylsalicylic acid at 37°C for 15 min. EDTA (5 mM) was added to PRP and it was centrifuged at 600 g for 10 min. Cells were washed in buffer A (20 mM HEPES, 138 mM NaCl, 2.9 mM KCl, 1 mM MgCl_2_, 0.36 mM NaH_2_PO_4_, 1 mM EGTA, supplemented with 5 mM glucose. Platelets were finally resuspended in buffer B (pH 7.4), which was same as buffer A, but without EGTA. Final cell count was adjusted to 2–4 × 10^8^/ml with Multisizer 4 (Beckman Coulter). All steps were carried out under hygienic conditions, and precautions were taken to maintain the cells in resting state.

### Surface Plasmon Resonance

Interaction between PrP(106–126) and fibrinogen from human plasma was examined using BIAcoreT200 system (GE Healthcare Life Sciences, United States). PrP(106–126) was covalently immobilized on C1 sensor chip via amine coupling up to 2000 resonance units (RU). To arbitrate binding affinity (K_D_), a binding kinetics assay was performed at 25°C by perfusing increasing concentrations of fibrinogen (0.02–3.13 µM, in 10 mM HEPES, 150 mM NaCl, 0.05% Tween 20, pH 7.4) over PrP(106–126)-coated C1 sensor chip at flow rate of 30 µl/min. The resulting sensorgram was analyzed, and K_D_ was calculated with BIAcore T200 evaluation software (V. 2.0), using a steady-state affinity binding model.

### Measurement of Cytosolic Free Ca^2+^ in Platelets

PRP was incubated with Fura-2-AM (2 µM) at 37°C for 45 min. Fura-2-loaded platelets were washed and resuspended in buffer B at 10^8^ cells/ml. Fluorescence was recorded in 400 µl aliquots of platelets under nonstirring conditions at 37°C using Hitachi fluorescence spectrophotometer (model F-2500). Fura-2 was excited at 340 and 380 nm, and the emission wavelength was set at 510 nm. Changes in intracellular free Ca^2+^ [Ca^2+^]_i_ were monitored from the fluorescence ratio (340 nm/380 nm) using the Intracellular Cation Measurement Program in FL solutions software as described earlier (Gautam et al., 2019). F_max_ was determined by lysing platelets with 250 µM digitonin in presence of saturating CaCl_2_. F_min_ was determined by adding 12 mM EGTA [Ca^2+^]_i_ was calibrated according to derivation of Grynkiewicz et al. (1985).

### Extracellular Vesicle (EV) Release From Platelets

Washed platelets were incubated with prion peptide (20 µM) in the presence or absence of fibrinogen (2 mg/ml) for 15 min at 37°C without stirring. Cells were pelleted by centrifugation at 4600 g for 5 min, and EVs were acquired in supernatant. EVs were fixed with 2% paraformaldehyde (PFA) and characterized with the Nanoparticle Tracking Analyzer (NTA) where a beam from a solid-state laser source (635 nm) was allowed to pass through the sample. Light scattered by rapidly moving particles in suspension in Brownian motion at room temperature was observed under 20X microscope. This revealed hydrodynamic diameters of particles, calculated using the Stokes Einstein equation, within range of 10 nm to 1 µm and concentration between 10^7^–10^9^/ml. The average distance moved by each EV in *x* and *y* directions were captured with CCD camera (30 frames per second) attached to the microscope. Both capture and analysis were performed using NanoSight LM10 (Malvern) and NTA 2.3 analytical software, which provides an estimate of the particle size and counts in sample as described earlier (Gautam et al., 2019; Kailashiya et al., 2019). Calcium ionophore A23187 (1 μM) was used as positive control to release EVs from platelets.

### Platelet Calpain Activity Assay

Calpain activity was assayed as described earlier (Mallick et al., 2015). Washed human platelets were treated with PrP(106–126) (20 µM) in the presence or absence of fibrinogen (2 mg/ml) in 96-well plates for 10 min followed by incubation with *t*-butoxycarbonyl-LeuMetchloromethylcoumarin (10 µM) for 30 min. Subsequently, cellular fluorescence was quantified with fluorescence multimode microplate reader (BioTek, model Synergy H1) at 37°C (excitation, 351 nm; emission, 430 nm).

### Immunoblotting

Human platelets were incubated with PrP(106–126), in the presence or absence of fibrinogen (2 mg/ml) and calcium ionophore A23187 (1 µM) as indicated, followed by heating in Laemmli lysis buffer. Platelet proteins were separated on 10% sodium dodecyl sulfate polyacrylamide gel electrophoresis (SDS-PAGE) and electrophoretically transferred to a PVDF membrane, using a semidry blotting system (BioRad). Membrane was blocked with 5% nonfat dry milk in Tris-buffered saline (10 mM Tris-HCl and 150 mM NaCl, pH 8.0) containing 0.05% Tween-20 (TBST) for 1 h at room temperature. The blot was incubated overnight with anti-talin antibody (1:5000 dilution), followed by three washings with TBST for 5 min each. The membrane was placed in HRP-labeled anti-mouse IgG diluted in blocking buffer (1:10,000) for 1 h. The blot was similarly washed, and antibody binding was detected using enhanced chemiluminescence detection kit (Millipore). Images were acquired on a multispectral imaging system (BioSpectrum 800 Imaging system, UVP) and quantified using VisionWorks LS software (UVP) (Gautam et al., 2019).

### Cell Culture

Neuroblastoma SH-SY5Y cells were grown in DMEM/F12 media supplemented with 10% heat-inactivated fetal bovine serum, 1% MEM nonessential amino acids, and 1% antibiotic-antimycotic solution (penicillin, streptomycin, and amphotericin) solution at 37°C in a 95% humidified air-5% CO_2_ incubator (Thermo). Cells were cultured in flasks or plates for 24 h till they reached 80% confluency and washed three times with culture medium. To evaluate the protective effect of fibrinogen, cells were incubated with PrP(106–126) (20 µM) in the presence or absence of fibrinogen (2 mg/ml) for 48 h. After respective treatments, cells were detached with trypsin (0.25%)/EDTA (1 mM) and harvested for analysis. Cells that underwent less than 15 passages were used in our study and all experiments were repeated several times with different batches of cells. For flow cytometry, cells were cultured in six-well plates, whereas 96-well plates were employed for cell viability assays. Serum-starvation and vehicle did not affect viability of control samples.

### Cell Viability Assay

SH-SY5Y cells at a density of 1 × 10^6^ cells per ml were cultured in 96-well plates for 24 h, followed by 48 h incubation with PrP(106–126) in the presence or absence of fibrinogen. Cell viability was measured employing 3-(4,5-dimethylthiazol-2-yl)-2,5-diphenyltetrazolium bromide (MTT) assay as earlier described (Soundararajan et al., 2008). MTT (100 µl) was added to each well to the final concentration of 0.5 mg/ml and incubated for 4 h at 37°C. The formazan crystals generated were thoroughly dissolved in 100 µl DMSO, and the absorbance was measured at 540 nm using microplate reader.

### Measurement of Mitochondrial Membrane Potential (ΔΨm)

SH-SY5Y cells were treated with prion and fibrinogen as described above, followed by incubation with MitoTracker Red dye (500 nM) for 45 min at RT. MitoTracker Red accumulates in live cell mitochondria that are negatively charged and is employed as a measure of mitochondrial function. Mitochondrial membrane potential of harvested cells was analyzed on a flow cytometer (FACSCalibur, BD Biosciences). Fluorescence data from each sample were collected using logarithmic amplification for 500 events in platelet gate (FL2 channel) and analyzed using CellQuest Pro Software.

### Confocal Microscopy

Cultured SH-SY5Y cells (10^5^ cells in 1 ml) were fixed with an equal volume of chilled paraformaldehyde (2%) and incubated for 20 min on ice. Fixed cells were centrifuged at 200 g for 5 min and resuspended in PBS, followed by permeabilization with 0.1% Triton-X-100 for 2 min. Cells were stained with propidium iodide (0.5 mM) and phalloidin-FITC (2.5 μM). Five images from random fields in each well were visualized under a laser scanning confocal microscope (Zeiss, model LSM 700) with ×63 objective and 1 AU pinhole size. Images were acquired and analyzed using ZEN imaging software.

For fibrinolysis study, clot formation was induced on the coverslip by mixing fibrinogen solution (0.5 mg/ml in PBS containing 10% Alexa Fluor 488-conjugated fibrinogen) with calcium chloride (2 mM) and thrombin (1 U/ml) for 1 h, in either the presence or absence of PrP(106–126). Clots were lysed with plasminogen (200 nM) and tissue plasminogen activator (0.5 µg/ml) for 5 min and were fixed with 2% paraformaldehyde for 20 min. Images from random fields were acquired before and after fibrinolysis and analyzed using ImageJ software (National Institutes of Health).

### Clot Formation and Lysis Assays

Clot formation was induced by the addition of calcium chloride (2 mM) and thrombin (1U/ml) to 100 µl aliquots of fibrinogen solution (0.5 mg/ml in PBS containing 10% Alexa Fluor 488-conjugated fibrinogen) in the presence or absence of prion peptide (20 µM). Turbidity of the clot increased with fibrin polymerization that was recorded as a change in absorbance at 640 nm at 5-min intervals using a multimode microplate reader. The plateau of the turbidity curve denoted complete clot formation. For clot lysis assay, plasminogen (200 nM) and tissue plasminogen activator (0.5 µg/ml) were added to fluorescent fibrin clots generated in microplate wells as described above and incubated for 10 min at 37°C to allow clot lysis. Supernatant (containing fluorescently labeled fibrin degradation products) was collected from each well and fluorescence (excitation, 488 nm; emission, 530 nm) was measured using a fluorescence microplate reader.

### Scanning Electron Microscopy

Fibrin clots were prepared on glass slides by dropping 20 µl mixture of fibrinogen (1 mg/ml) and CaCl_2_ (2 mM) in PBS with or without prion peptide (20 µM), followed by addition of 1 U/ml thrombin to initiate polymerization. After 1 h, jelly-like clots on the slides were gently washed with Milli Q water, allowed to dry, coated with gold using a sputter coater (Quorum Technologies, model SC7620) and examined under a scanning electron microscope (Zeiss, model EVO 18). A secondary electron detector and energy dispersive spectrometer were used for morphological studies and elemental analysis, respectively.

### Statistical Methods

All statistical analyses were performed using GraphPad Prism 8 software. One-tailed Student’s *t*-test (for two groups) or one-way analysis of variance (ANOVA) (for three or more groups) was employed for evaluating significance in difference of means between groups, and values of *p* < .05 were considered significant.

## Results

### Interaction Between PrP (106–126) and Fibrinogen

**FIGURE 1:**
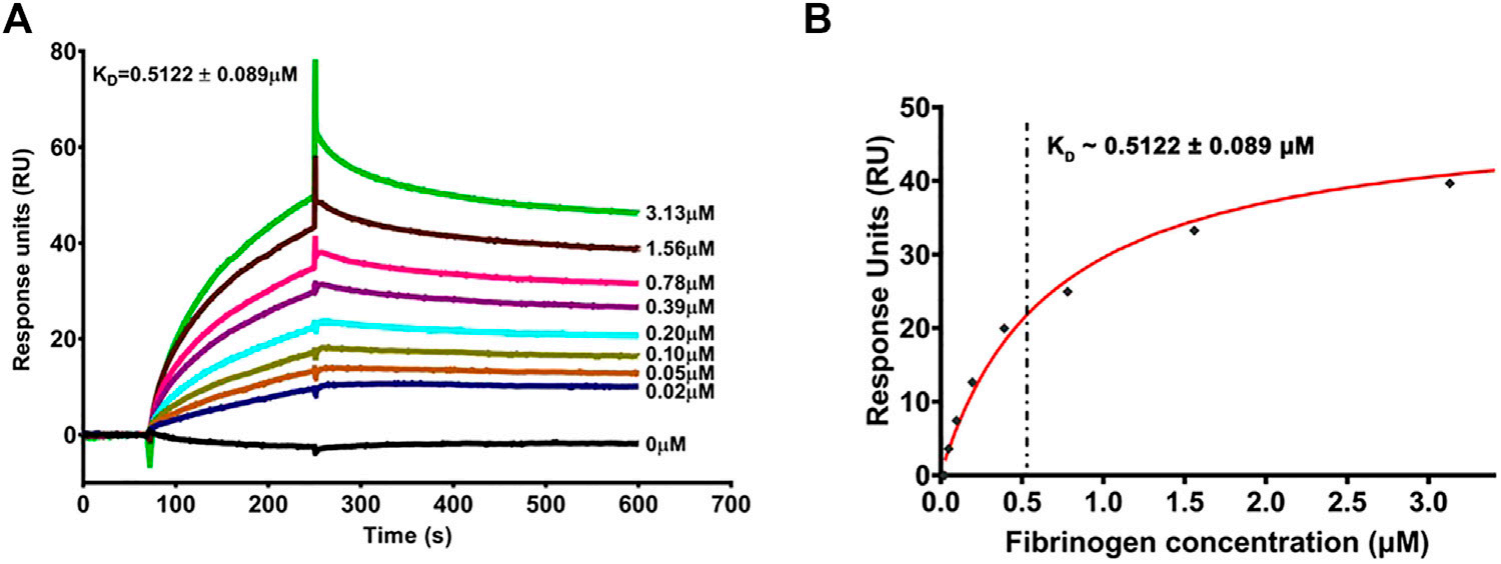
Surface plasmon resonance analysis of dose-dependent interaction between PrP(106–126) and human plasma fibrinogen. **(A)** Sensorgram showing dose-dependent binding between fibrinogen (0.02–3.13 μM) and PrP(106–126)-coated CM5 sensor chip. The values against sensorgram tracings in panel A represent concentrations of fibrinogen. **(B)** The steady-state curve-fitting model showing the binding affinity constant (K_D_) at ∼ 0.5122 ± 0.089 μM. Figure is representative of ≥3 different experiments.

The amino acid sequence 106–126 from prion proteins, PrP(106–126), is highly amyloidogenic and is implicated in the rise in platelet intracellular calcium and microparticle shedding (Brown et al., 1997; Mallick et al., 2015) as well as prion-mediated neurotoxicity (Chiesa, 2015). As amyloid β, another amyloidogenic peptide rich in β-pleated sheets that elevates cytosolic calcium in platelets (Mattson et al., 1992), is reported to interact with fibrinogen β-chain (Ahn et al., 2010; Zamolodchikov and Strickland, 2012), we asked whether prion, too, has affinity toward fibrinogen. Strikingly, the binding kinetic assay performed employing SPR revealed high-affinity binding between PrP(106–126) and fibrinogen in a dose-dependent manner (Figure 1). The interaction was augmented with an increase in fibrinogen concentration from 0.02 to 3.13 μM with the binding affinity constant, K_D_, at 0.5122 ± 0.089 μM**.**

### Fibrinogen Prevented PrP(106–126)-Induced Rise in Intracellular Ca^2+^ and Shedding of Extracellular Vesicles From Platelets

**FIGURE 2:**
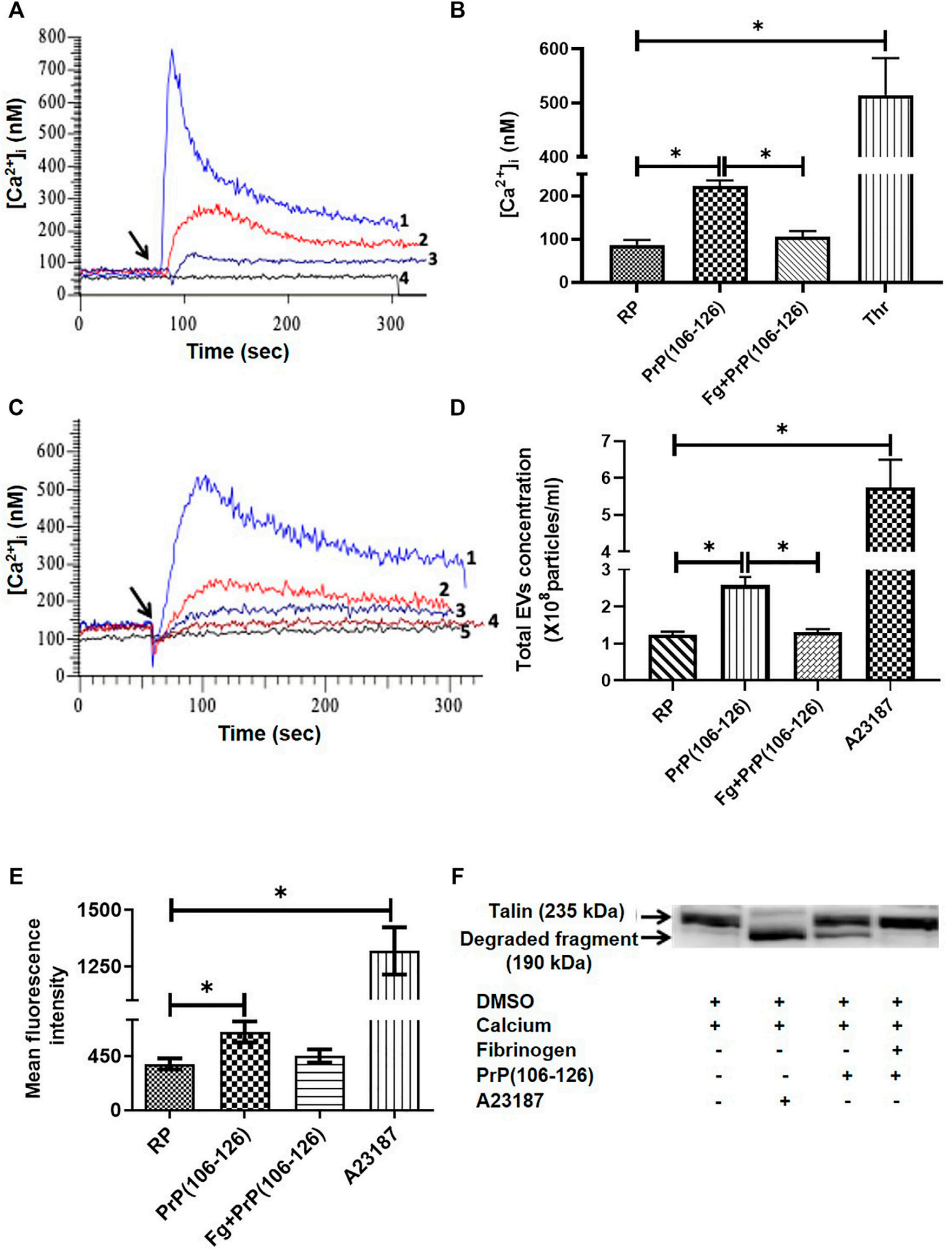
Fibrinogen attenuated PrP(106–126)-induced rise in free intracellular Ca^2+^, EV release, calpain activity, and talin degradation in human platelets. **(A)** Tracings 1 to 4 represent platelets treated with thrombin (0.5 U/ml), PrP(106–126) (20 µM), fibrinogen (1 mg/ml) plus PrP(106–126), and vehicle, respectively. Ca^2+^ (1 mM) was added in all samples. **(B)** Graphic presentation of corresponding values of panel A in bars. **(C)** Tracings 1 to 4 represent platelets treated with PrP(106–126) (20 µM) alone or in the presence of 0.5 mg/ml, 1 mg/ml, and 2 mg/ml fibrinogen, respectively. Tracing 5, vehicle-treated platelets. Ca^2+^ (1 mM) was added in all samples. **(D)** Extracellular vesicle shedding from PrP(106–126) (20 µM)-treated platelets in the presence or absence of fibrinogen (2 mg/ml) characterized with the Nanoparticle Tracking Analyzer. A23187 (1 μM) was used as a positive control. **(E)** Calpain activation in PrP(106–126) (20 µM)-treated platelets assayed in the presence or absence of fibrinogen (2 mg/ml). A23187 was used as a positive control. **(F)** Immunoblot representing degradation of talin (235 kDa) to its proteolytic fragment (190 kDa). Platelets were treated with different reagents as indicated. Figures are representative of ≥3 independent experiments (mean ± SEM, *p* < .05).

The basal level of intracellular Ca^2+^ in human platelets was maintained at 72.82 ± 28.23 nM (mean ± SEM), which soared to 152.9 ± 83.53 nM (mean ± SEM) in the presence of PrP(106–126) (20 µM) and 1 mM Ca^2+^ (Figures 2A–C). Strikingly, pretreatment of platelets with fibrinogen at concentrations ranging from 0.5 to 2 mg/ml, attenuated prion-induced rise in [Ca^2+^]_i_ in a dose-dependent manner (Figure 2B). Prion (20 µM) also prompted extensive shedding of extracellular vesicles from human platelets, which was completely thwarted upon prior exposure of platelets to fibrinogen (2 mg/ml) (Figure 2D). Calcium ionophore A23187 (1 μM) was employed as positive control.

### Fibrinogen Prevented PrP(106–126)-Induced Rise Calpain Activity and Talin Proteolysis in Human Platelets

Treatment with PrP(106–126) (20 µM) in the presence of 1 mM calcium elicited rise in activity of the platelet thiol protease calpain (by 66.60% ± 19.6), associated with the proteolysis of talin, a calpain substrate. When platelets were preincubated with fibrinogen (2 mg/ml) followed by exposure to prion, there was significant drop in calpain activity (by 30%) to nearly resting levels (Figure 2E). Fibrinogen also completely prevented prion-mediated cleavage of talin in platelets as suggested from disappearance of its 190 kDa proteolytic product (Figure 2F). As expected, treatment with the calcium ionophore A23187 (1 µM) resulted in significant enhancement in calpain activity in platelets associated with complete proteolysis of talin.

### Fibrinogen Protected SH-SY5Y Neuroblastoma Cells From PrP(106–126)-Induced Cytotoxicity

**FIGURE 3:**
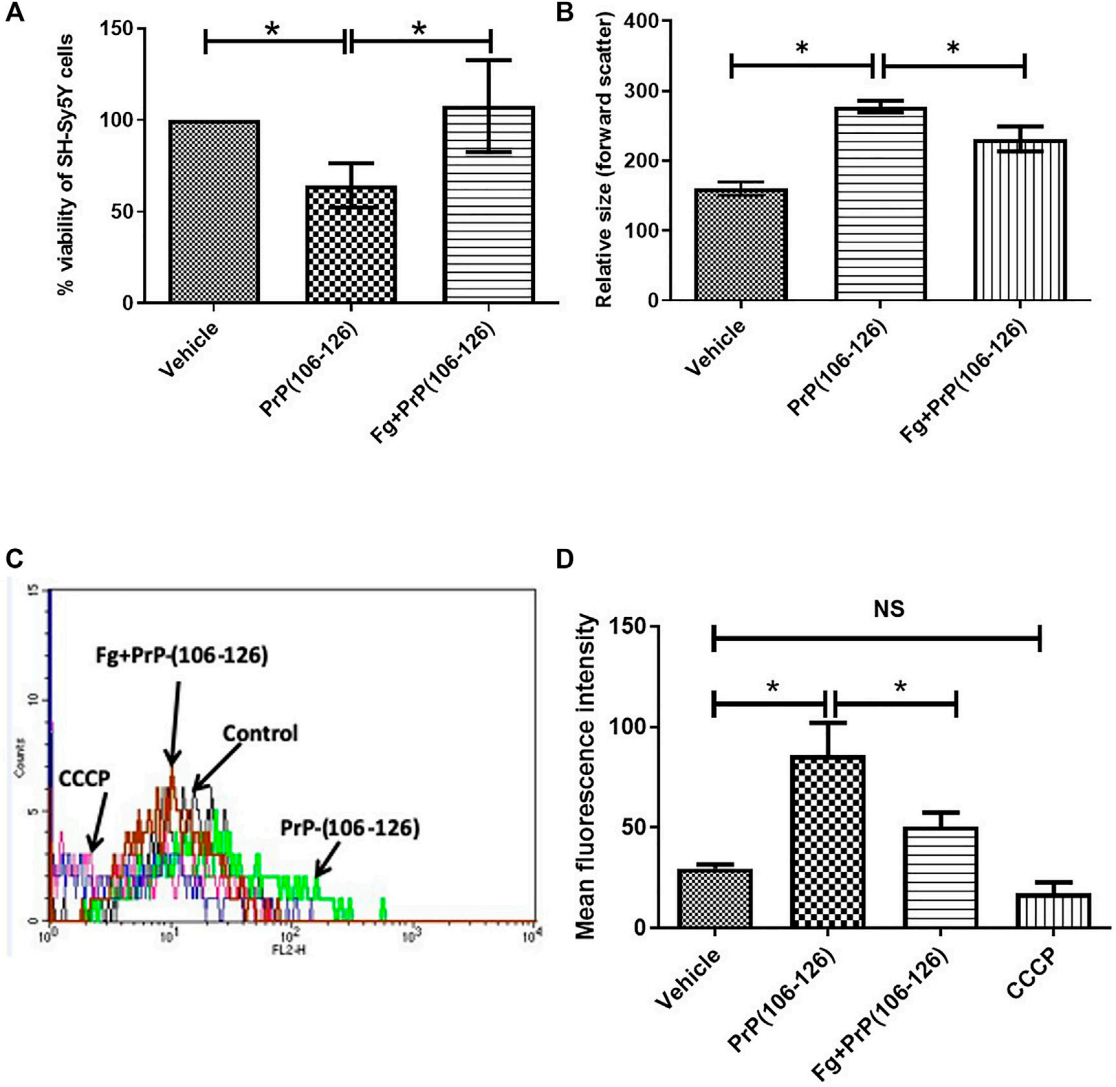
Fibrinogen prevented toxicity of prion peptide on SH-SY5Y neuronal cells. **(A)** Measurement of viability of SH-SY5Y cells by MTT assay following PrP treatment in the presence or absence of fibrinogen. **(B)** Flow cytometry of neuronal cells treated with PrP(106–126) in the presence or absence of fibrinogen. **(C,D)** Measurement of mitochondrial transmembrane potential by MitoTracker Red staining in prion peptide-treated SH-SY5Y neuronal cells in absence or presence of fibrinogen. Figures are representative of ≥3 independent experiments (mean ± SEM, *p* < 0.05).

Incubation with PrP(106–126) instigated a sharp decline in viability of neuroblastoma cell lines. From the MTT assay number of viable cells was found to be 53.28% less in PrP-treated sample, which was completely restored when cell lines were preincubated with fibrinogen (Figure 3A), suggestive of a protective effect of fibrinogen on prion-mediated neurotoxicity. Interestingly, exposure to PrP amplified forward scatter (FSC) in flow cytometry, indicative of cell size, by 73.28% ± 8.34, which was abrogated significantly (by 16.34% ± 17.85) upon prior treatment with fibrinogen (Figure 3B). Prion peptide, too, brought about hyperpolarization of mitochondrial transmembrane potential (ΔΨm) in MitoTracker Red-stained platelets to the extent of 2.9-fold. The rise in ΔΨm was attenuated by 1.7-fold on preincubation of cells with fibrinogen (Figures 3C,D).

### Prion-Induced Dysmorphic Growth of SH-SY5Y Cells Was Prevented by Fibrinogen

**FIGURE 4:**
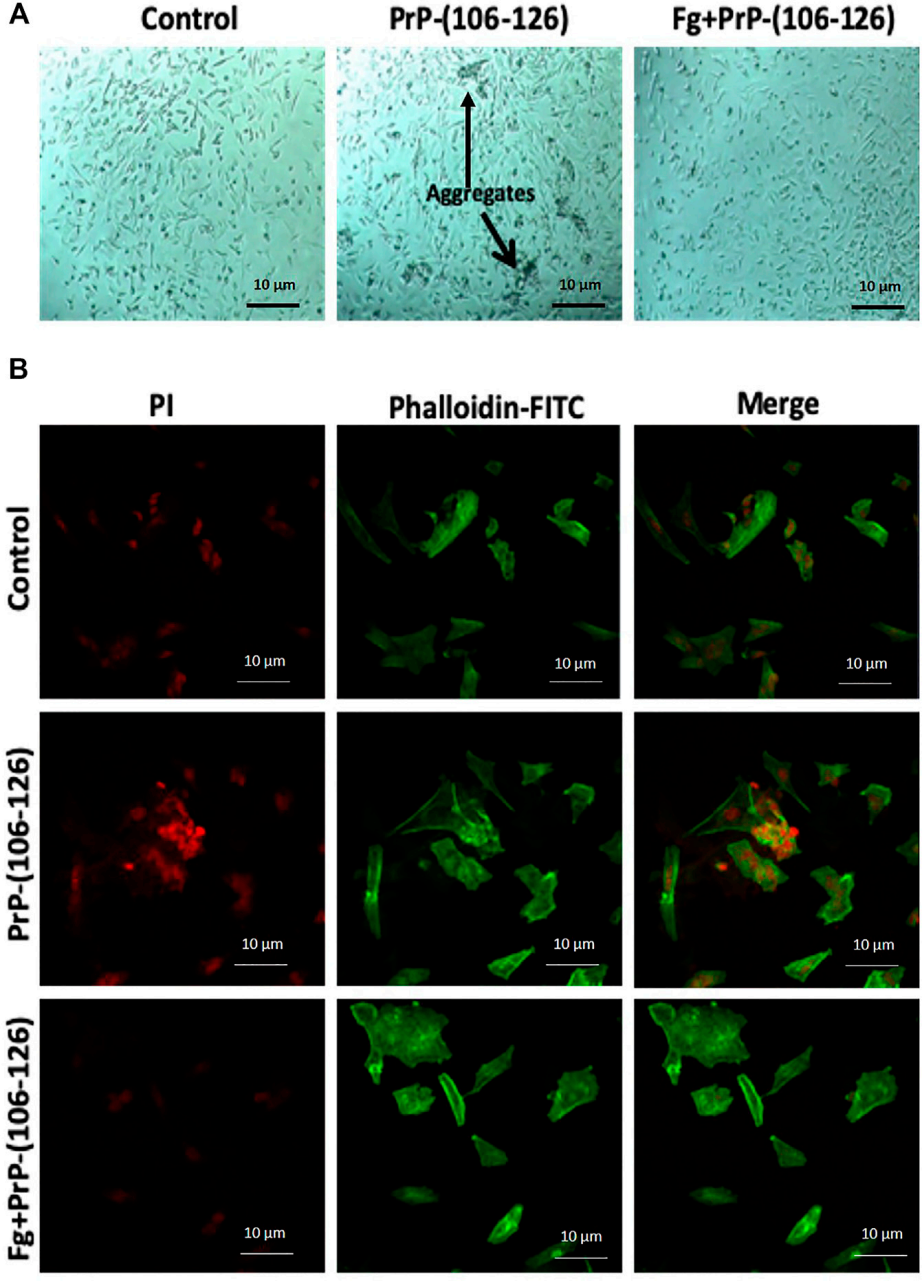
Fibrinogen diminished PrP(106–126)-prompted dysmorphic growth of SH-SY5Y cells. **(A)** SH-SY5Y cells, grown in culture media in presence of PrP and/or fibrinogen as indicated, were observed under phase contrast microscope. Arrows indicate cell aggregates. **(B)** SH-SY5Y cells, fluorescently labeled with propidium iodide (red) and phalloidin-FITC (green), were cultured in presence or absence of PrP and fibrinogen as indicated and observed under confocal microscope. Images are representative of five different fields each from three independent experiments.

SH-SY5Y cell line was incubated with PrP(106–126) in the absence or presence of fibrinogen, stained with propidium iodide (which stains nucleic acid) as well as phalloidin-FITC (which stains actin filaments), and morphology was examined. Phase contrast microscopy demonstrated abundant formation of cell aggregates upon prion treatment (Figure 4A). Remarkably, prion-treated stained cells, too, exhibited the presence of clustered nuclei when examined under a confocal microscope (Figure 4B). Preincubation with fibrinogen prevented the cells from forming aggregates as well as nuclear clusters (Figures 4A,B, respectively). Thus, fibrinogen preserved the normal morphology of neuronal cells, protecting them from toxic effects of prion peptide.

### PrP(106–126) Augments Fibrin Clot Formation, Alters Clot Structure, and Impairs Fibrinolysis

**FIGURE 5:**
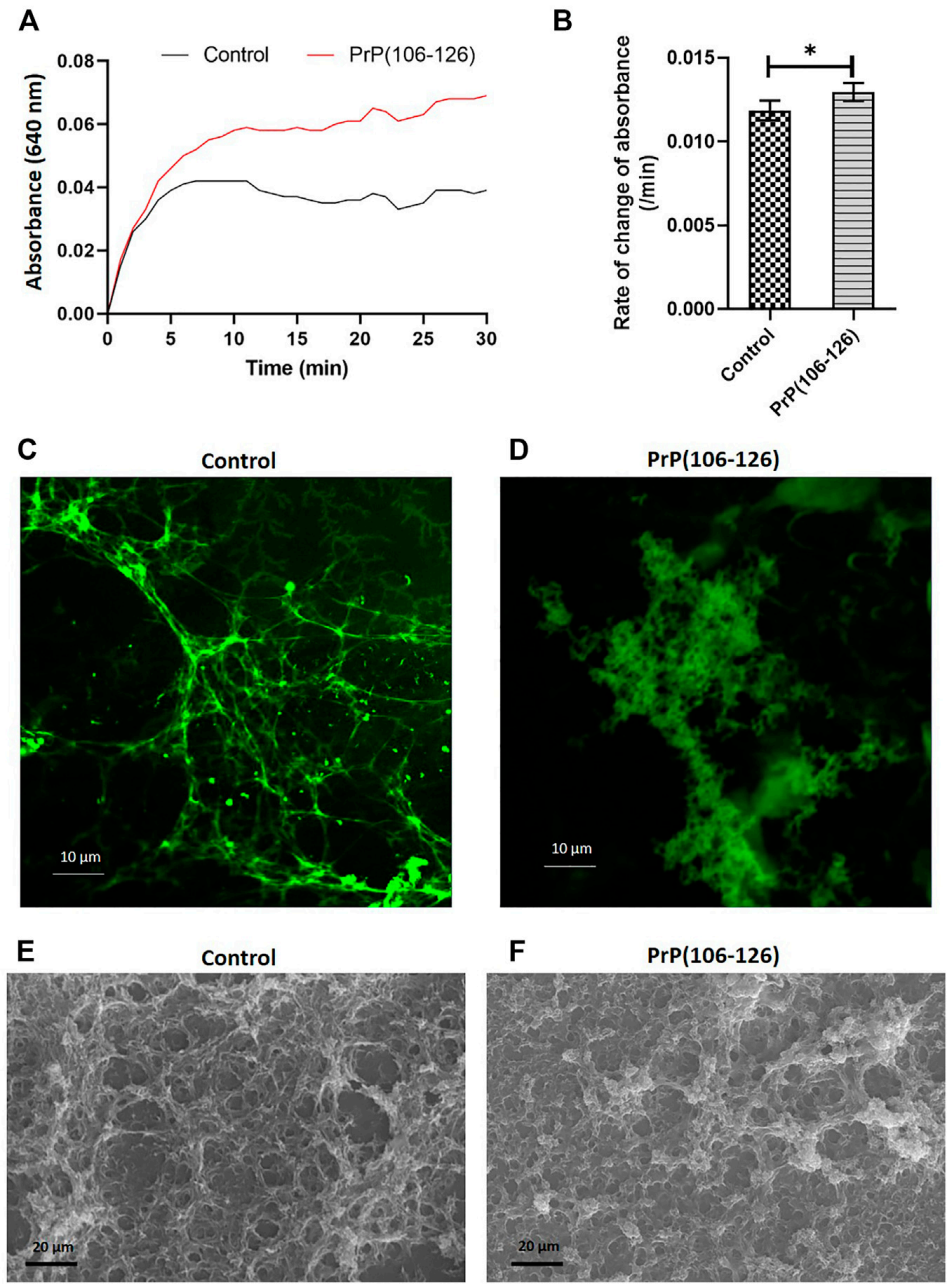
PrP(106–126) augments fibrin clot formation and alters clot structure. **(A)** Clot formation was induced by addition of thrombin to fibrinogen in the presence or absence of PrP. Turbidity of the clot was recorded as the change in absorbance at 640 nm at 5 min intervals. **(B)** Corresponding bar diagram representing rate of change of absorbance per minute (calculated by subtracting final absorbance value from the initial value and dividing by total duration, 30 min). **(C)** and **(D)** Confocal microscopy images of fluorescent fibrin clots formed in the absence **(C)** or presence **(D)** of PrP. **(E)** and **(F)** Scanning electron microscopy images of fibrin clot formed in the absence **(E)** or presence **(F)** of PrP. Figures are representative of ≥3 independent experiments (mean ± SEM, *p* < 0.05).

We next sought to determine if interaction between PrP(106–126) and fibrinogen would impact fibrin clot formation that is central to the process of hemostasis. Fibrin was allowed to be generated from the thrombin-mediated proteolytic cleavage of fibrinogen, which quickly polymerized into an insoluble clot. The kinetics of clot formation were analyzed by a turbidimetry-based assay, which exhibited a plateau within 5 min of thrombin addition. Notably, the presence of PrP in the incubation mix significantly amplified the extent of clot generated at each time point, reflective of seminal contribution of prion in augmenting the process of fibrin polymerization and clot formation (Figure 5A).

To determine the effect of PrP on clot structure, clots were allowed to form on coverslips employing 10% Alexa Fluor 488-conjugated fibrinogen and were examined under a confocal microscope. In the control sample, fibrils of fibrin so generated presented a uniform mesh-like structure. Contrasting this, PrP-treated samples presented clots as abundant, amorphous aggregates that were interspersed within the fibrin mesh (Figures 5C,D). The above observations were further supported by scanning electron microscopy (Figures 5E,F).

**FIGURE 6:**
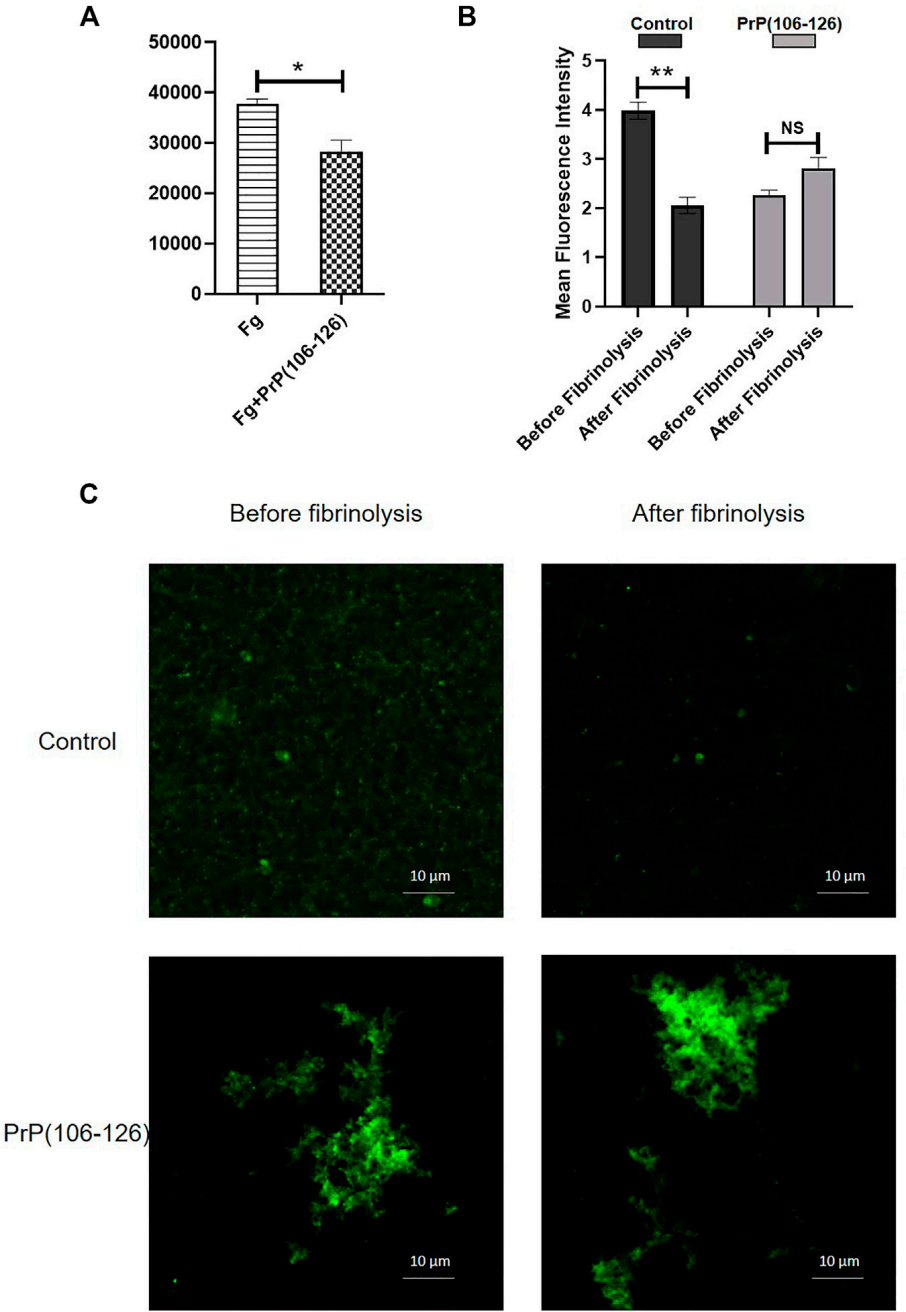
**(A)** Fibrinolysis measured from the extent of release of fluorescent degradation products from fibrin clots treated with tPA plus plasminogen. **(B)** Mean fluorescence intensity of fibrin clots treated with tPA plus plasminogen for 5 min, followed by confocal microscopy. Images were analyzed using ImageJ software. **(C)** Confocal images showing the effect of PrP(106–126) on plasmin-mediated lysis of fluorescently labeled fibrin clots. Figures are representative of ≥3 independent experiments (mean ± SEM, *p* < 0.05).

Plasmin-mediated thrombolysis is a physiological process for removal of clots from the sites of lesions. As prion alters the clot structure, it may impact the accessibility of tissue plasminogen activator (tPA) into the clot interior. Prion is also known to form complexes with plasminogen (Fischer et al., 2000; Praus et al., 2003). Thus, we next examined the effect of PrP on lysis of fluorescent fibrin clots by measuring extent of release of fluorescent fibrin degradation products. tPA induced clot lysis in the presence of plasminogen, which was significantly prohibited in samples pretreated with PrP (Figure 6A). In keeping with this, confocal microscopic images of fluorescent clots treated with tPA and plasminogen exhibited complete lysis (Figure 6C, upper panel), and the presence of prion provided resistance to degradation, leading to clot stabilization (Figures 6B,C, lower panel).

## Discussion

It has earlier been demonstrated that β-amyloid peptide (Aβ) interacts with the C-terminus of the fibrinogen β chain (Ahn et al., 2010). PrP has significant content of a beta-sheet secondary structure similar to Aβ peptide (Ahn et al., 2010). An earlier study from our lab has proven that fibrinogen attenuates Aβ-induced platelet activation and neuronal toxicity (Sonkar et al., 2016). Thus, it was pertinent to ask whether fibrinogen could also bind to PrP and diminish PrP-mediated toxicity on platelets as well as neuronal cells.

SPR analysis provided conclusive evidence that PrP and fibrinogen interact with each other (Figure 1). As fibrinogen is fundamental to hemostasis and thrombosis, it encouraged us to further examine the significance of this interaction in platelets and neuronal cells. We earlier reported that PrP(106–126) (20 μM) provokes calcium entry from extracellular medium, leading to a rise in cytosolic calcium (Mallick et al., 2015). As PrP(106–126) interacts with fibrinogen, we next studied the effect of PrP(106–126) (20 μM) on washed human platelets in the presence of fibrinogen (2 mg/ml), which is close to its physiological level in plasma. We found that fibrinogen protected platelets from prion-mediated changes, including a rise in intracellular calcium (Figures 2A–C) and shedding of extracellular vesicles (Figure 2D).

Calpain, a thiol protease, is dependent on cytosolic calcium for its activity (Momeni, 2011). As fibrinogen abrogated the rise in platelet intracellular calcium ensued upon prion treatment, we asked whether fibrinogen could also restrain upregulation of calpain activity in these cells. Calpain proteolytic activity provoked by prion was found to be significantly impaired when platelets were preincubated with fibrinogen (Figure 2E). Cytoskeletal protein talin is a known calpain substrate (Kerstein et al., 2017) that provides structural stability to the cell (Roberts and Critchley, 2009; Chinthalapudi et al., 2018). As fibrinogen disallowed calpain activity, we examined talin expression in prion-treated platelets in the presence or absence of fibrinogen. Expectedly, fibrinogen completely averted prion-mediated talin degradation in platelets (Figure 2F). Platelets release EVs when challenged with physiological agonists, calcium ionophore, or under conditions of stress (Piccin et al., 2007; Yuana et al., 2013; Kerstein et al., 2017). We earlier demonstrated extensive shedding of EVs from PrP-treated platelets (Mallick et al., 2015), which was completely prohibited upon prior exposure of platelets to fibrinogen (Figure 2D).

**FIGURE 7:**
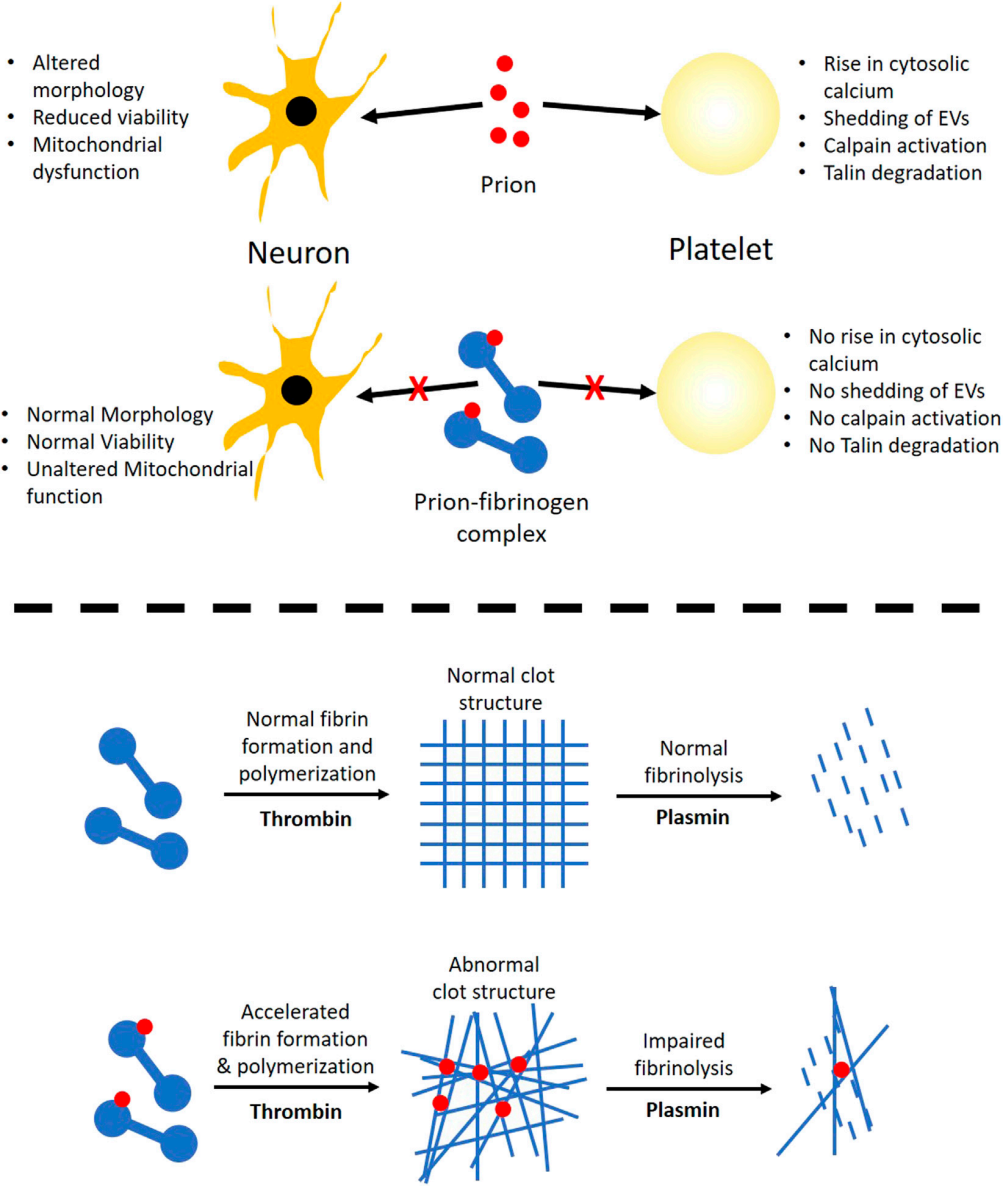
Scheme depicting the role of prion-fibrinogen complex in mitigation of prion-mediated platelet activation/neurotoxicity (upper panel), and modulation of fibrin clot dynamics (lower panel).

The hallmark of prion is that it is a self-perpetuating protein and has the ability to spread between cells and organisms. Cytoplasmic prion can spread during cell division and cell fusion. In mammals host-to-host horizontal transfer occur through environment and tissue barrier. Neuronal cells endocytose prion aggregates and transport them via acidic vesicles throughout cytoplasm, where it travels across the neuritic projections to reach the point of contact between two cells. The membrane-anchored PrPc gets converted to infectious PrPsc in prion-infected cells (Caughey et al., 2009). The prion peptide can remain associated with the membrane through glycophosphatidylinositol (GPI) anchor (Chiesa, 2015). Prion incites mechanical distortion of cell membranes adversely affecting membrane trafficking and leading to loss in cell viability (Caughey et al., 2009; Hu and Huang, 2013; Chiesa, 2015). We observed distinct morphological changes in prion-spiked neuronal cells, including abundant formation of cell aggregates and nuclear clusters, which were averted when cells were grown in the presence of fibrinogen (Figure 4). Hyperpolarization of mitochondrial transmembrane potential, a measure of cellular toxicity (Gergely et al., 2002; Perl et al., 2004), in PrP-treated neuronal cells was, too, mitigated by fibrinogen (Figures 3C,D). Consistent with this, a decline in neuronal cell viability upon exposure to PrP was completely restored when cell lines were preincubated with fibrinogen (Figure 3A). Taken together, the above observations strongly support a protective role of fibrinogen against prion-induced neurotoxicity, possibly by establishing fibrinogen-prion complex and, thus, restricting prion availability for cellular interaction and toxicity (Figure 7, upper panel).

Although our findings establish that fibrinogen curbs toxicity of PrP (106–126) through direct interaction with the prion peptide, fibrinogen itself is known to be a key player in thrombosis and hemostasis (Weisel and Litvinov, 2017; Kattula et al., 2017). Thus, it prompted us to ask if the complex of PrP with fibrinogen would, in turn, impact conversion of the latter into an insoluble clot and eventual fibrinolysis. Fibrinogen was allowed to be cleaved by thrombin to generate fibrin that quickly polymerized into the fibrin-rich mass. Notably, PrP (106–126) was found to accelerate thrombin-induced clot formation, which reflected the seminal contribution of prion in the process of fibrin polymerization. The architecture of the clot was also found to be distorted in PrP-treated samples with the abundant presence of amorphous aggregates interspersed within the fibrin mesh (Figure 5, C-F). Remarkably, interaction between amyloid β and fibrinogen was earlier reported to be present with an identical phenotype marked with potentiation of fibrinogen oligomerization (Ahn et al., 2010) and expression of structurally altered ‘amyloid β-influenced’ fibrin clots (Cortes-Canteli et al., 2010). Thus, the above observations point to a common mechanistic motif in modulation of thrombogenesis by β pleat-rich peptides such as amyloid β and prion.

Fibrin clots are eventually lysed by plasmin, which is generated from the proteolytic cleavage of plasminogen by tissue plasminogen activator (tPA). As PrP-influenced extensive structural changes in the clot could restrict access of tPA into the clot interior (Bailey et al., 2015) and adversely impact fibrin degradation, we next studied the effect of prion on clot lysis. Plasminogen in the presence of tPA induced fibrinolysis, which was significantly impeded in samples pretreated with PrP (Figure 6A), thus facilitating the persistence of the clot. Once prion protein is transferred pathologically in the central and peripheral nervous system, it is transported in the circulation by crossing the blood–brain barrier through absorptive endocytosis. Thus, it is tempting to hypothesize that the clot-stabilizing effect of the peptide would contribute to physiological hemostasis. Interestingly, amyloid β, too, is reported to delay fibrin clot lysis by altering fibrin structure (Zamolodchikov and Strickland, 2012), which further lends support to a shared mechanism between the β pleat-rich peptides in ensuring clot stability with thrombogenic consequences.

In conclusion, we report here that fibrinogen reverses PrP (106–126)-induced platelet activation and neurotoxicity while prion peptide, on the other hand, alters the morphology of the fibrin clot making it more resistant to lysis. The above observations provide useful insight toward developing pharmacological modulators, small molecules, or peptides mimicking PrP-binding sites on fibrinogen that may attenuate the adverse effects of prion on both platelets as well as neuronal cells.

## Data Availability

The data sets presented in this study can be found in online repositories. The names of the repository/repositories and accession number(s) can be found below: https://papers.ssrn.com/sol3/papers.cfm?abstract_id = 3928514.
