# Supplementary material for: Fibrinogen Mitigates Prion-Mediated Platelet Activation and Neuronal Cell Toxicity
Source: Front Cell Dev Biol. 2022 Mar 21;10:834016. doi: 10.3389/fcell.2022.834016 (PMC8977893; doi:10.3389/fcell.2022.834016)
Supplement: Supplementary file 1 [file DataSheet1.ZIP › Figure 2/Western blot of talin.pptx]

## Slide 1
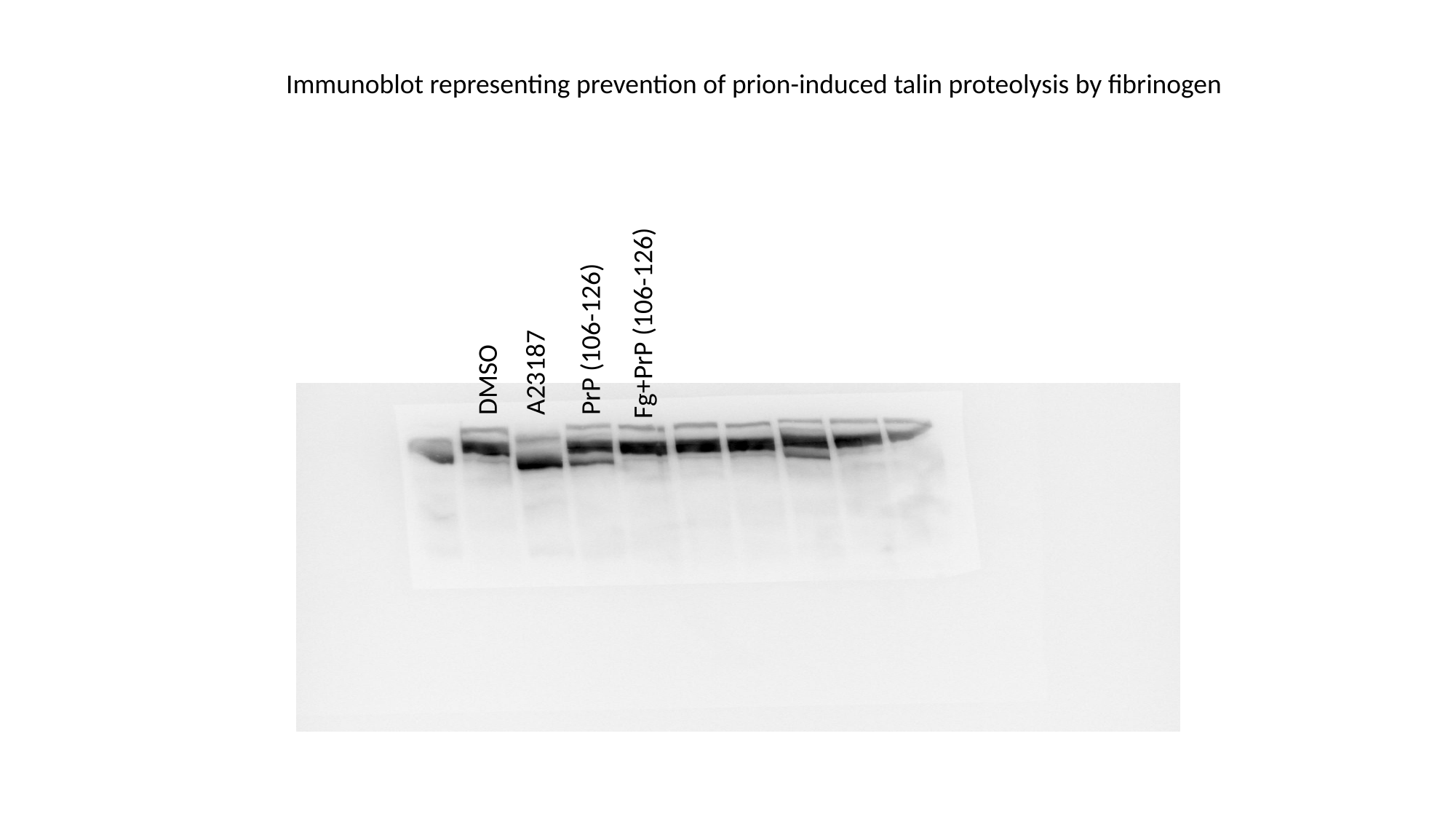

Immunoblot representing prevention of prion-induced talin proteolysis by fibrinogen
Fg+PrP (106-126)
PrP (106-126)
DMSO
A23187
