# Supplementary figures and images for: Fibrinogen Mitigates Prion-Mediated Platelet Activation and Neuronal Cell Toxicity
Source: Front Cell Dev Biol. 2022 Mar 21;10:834016. doi: 10.3389/fcell.2022.834016 (PMC8977893; doi:10.3389/fcell.2022.834016)

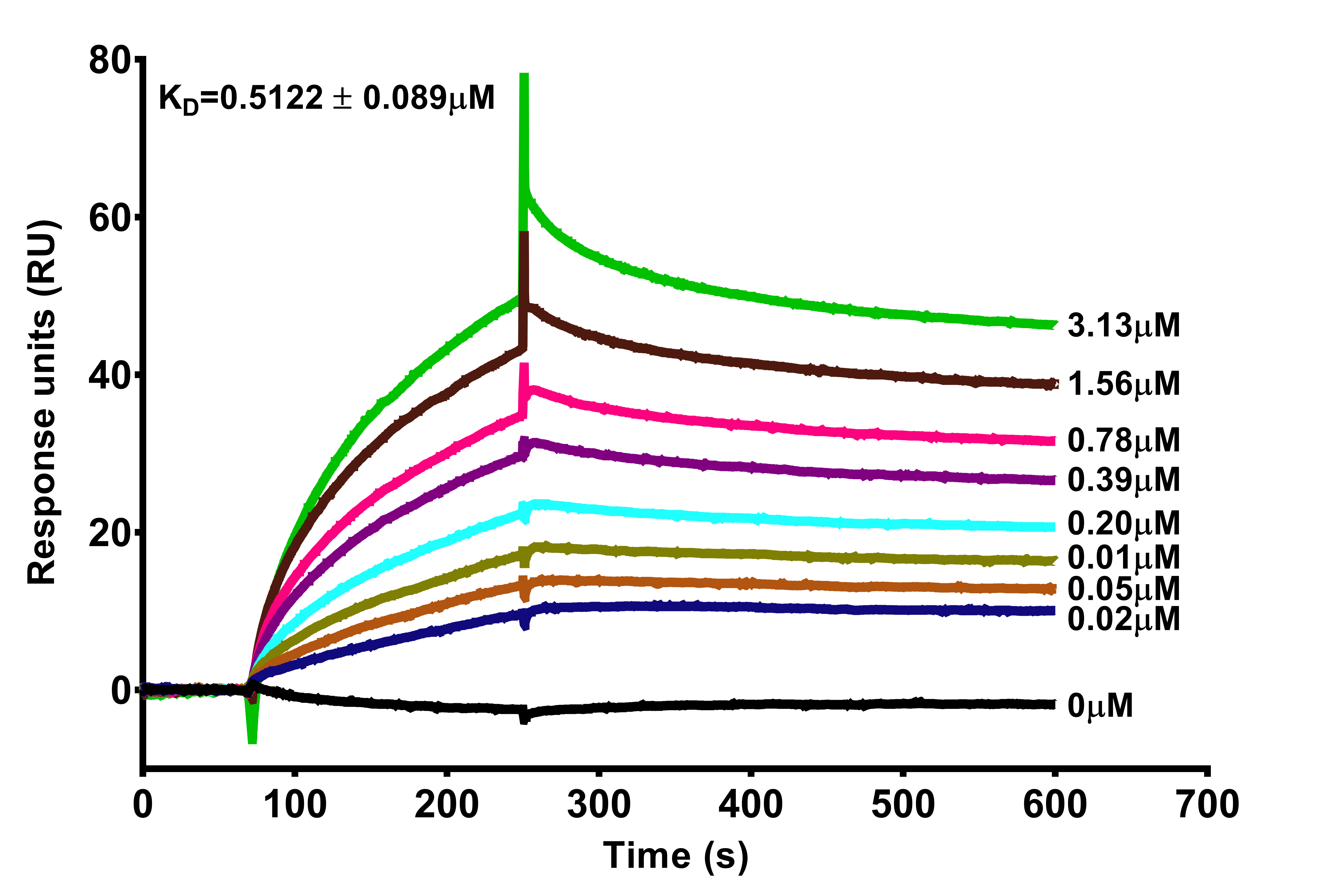

Supplement: Supplementary file 1 [file DataSheet1.ZIP › Figure 1/Sensogram showing binding of prion pep to Fibrinogen.tif]

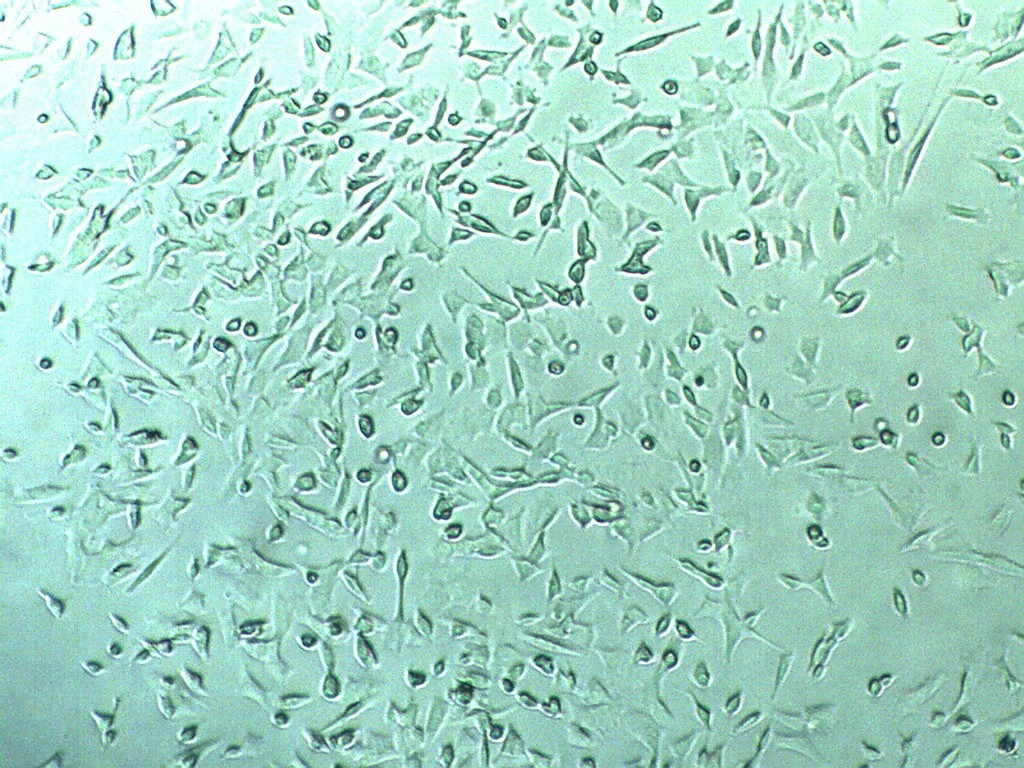

Supplement: Supplementary file 1 [file DataSheet1.ZIP › Figure 4/Control 1.jpg]

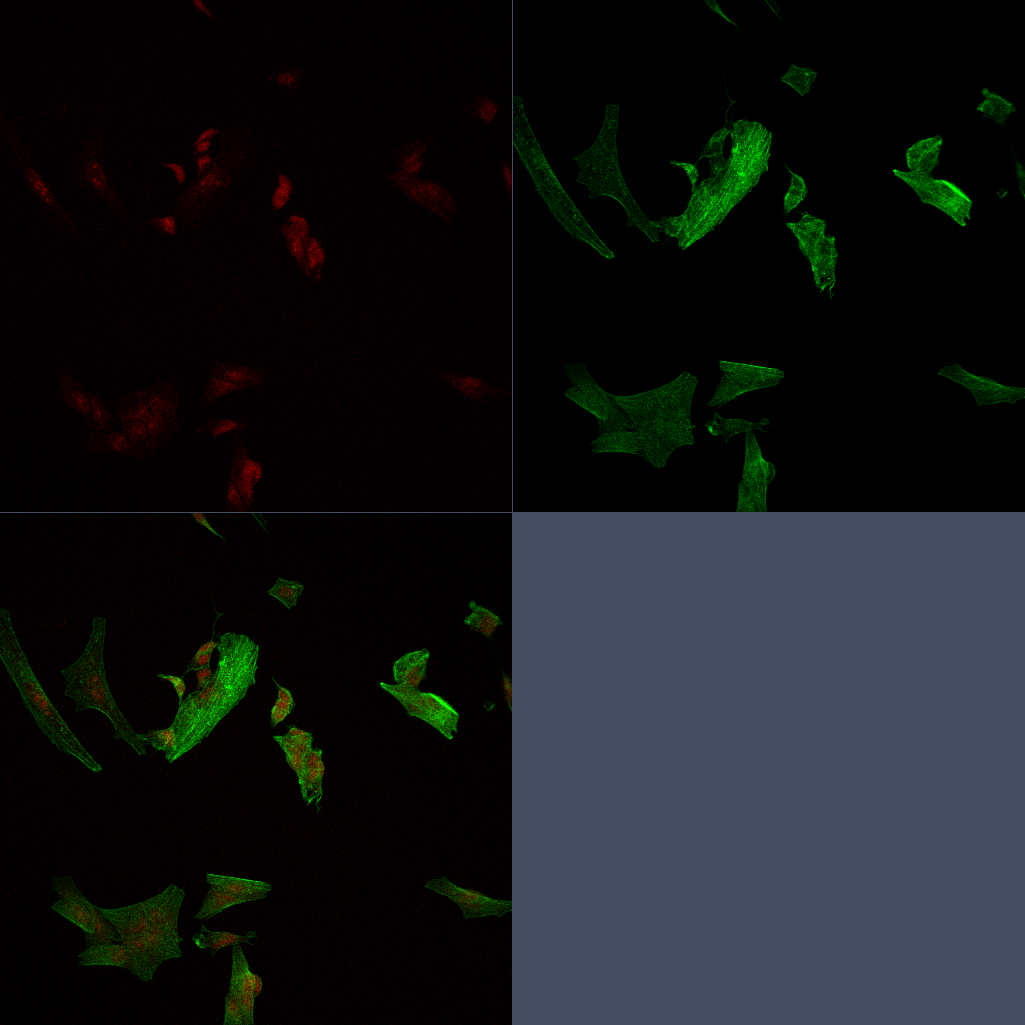

Supplement: Supplementary file 1 [file DataSheet1.ZIP › Figure 4/Control.jpg]

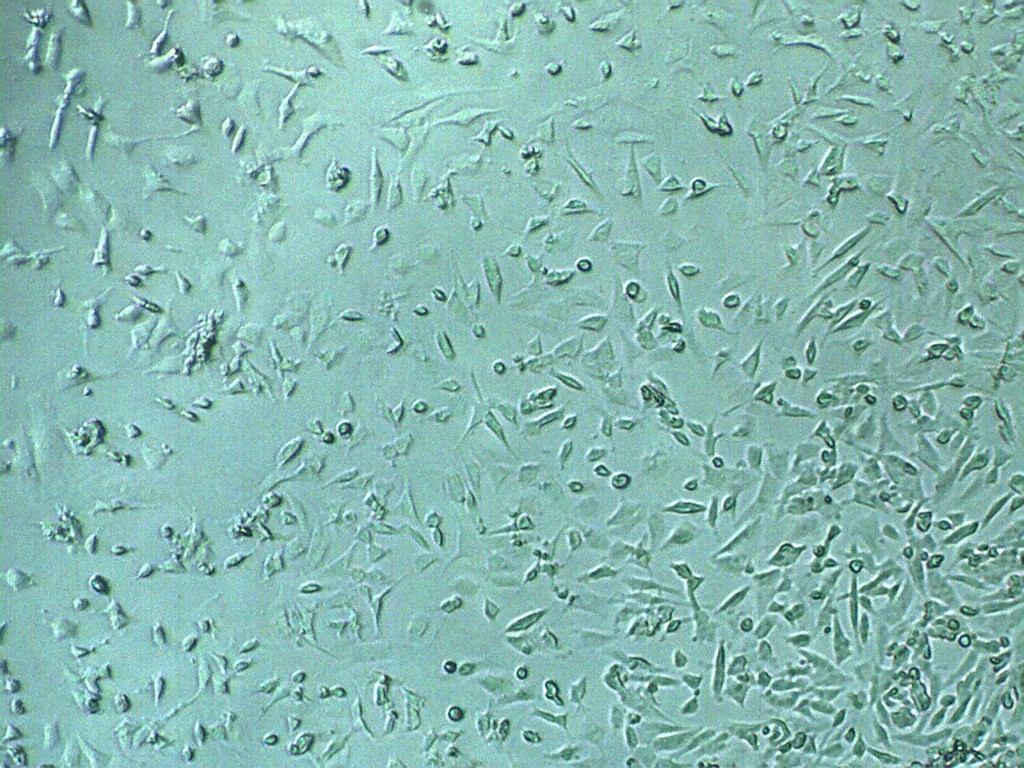

Supplement: Supplementary file 1 [file DataSheet1.ZIP › Figure 4/Fg+PrP (106-126).jpg]

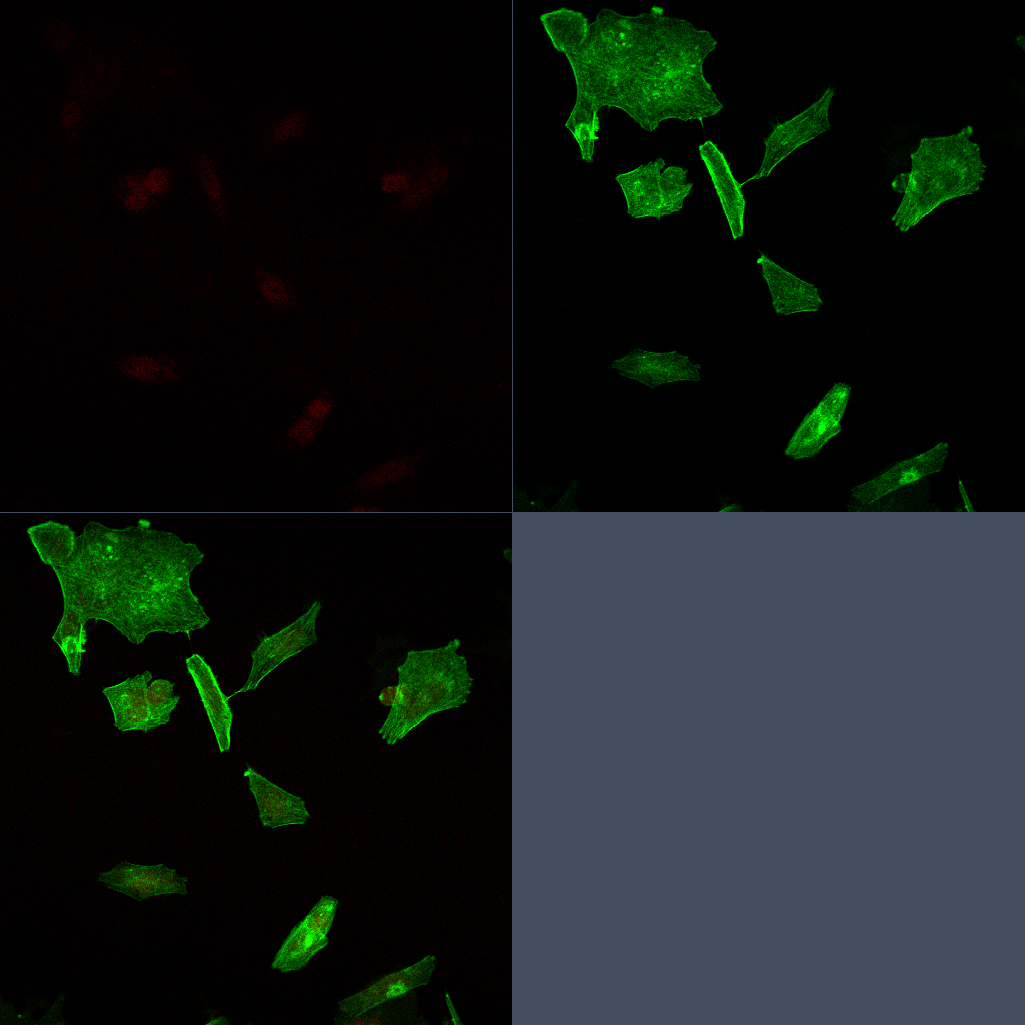

Supplement: Supplementary file 1 [file DataSheet1.ZIP › Figure 4/Fibrinogen+PrPs (106-126).jpg]

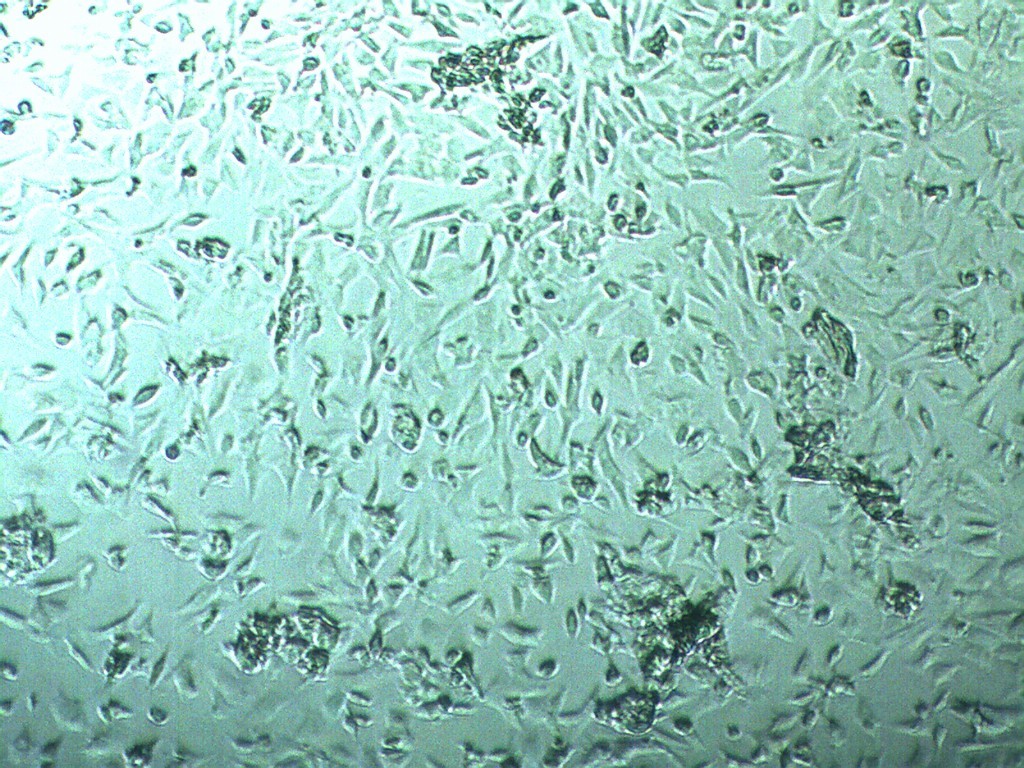

Supplement: Supplementary file 1 [file DataSheet1.ZIP › Figure 4/PrP(106-126).jpg]

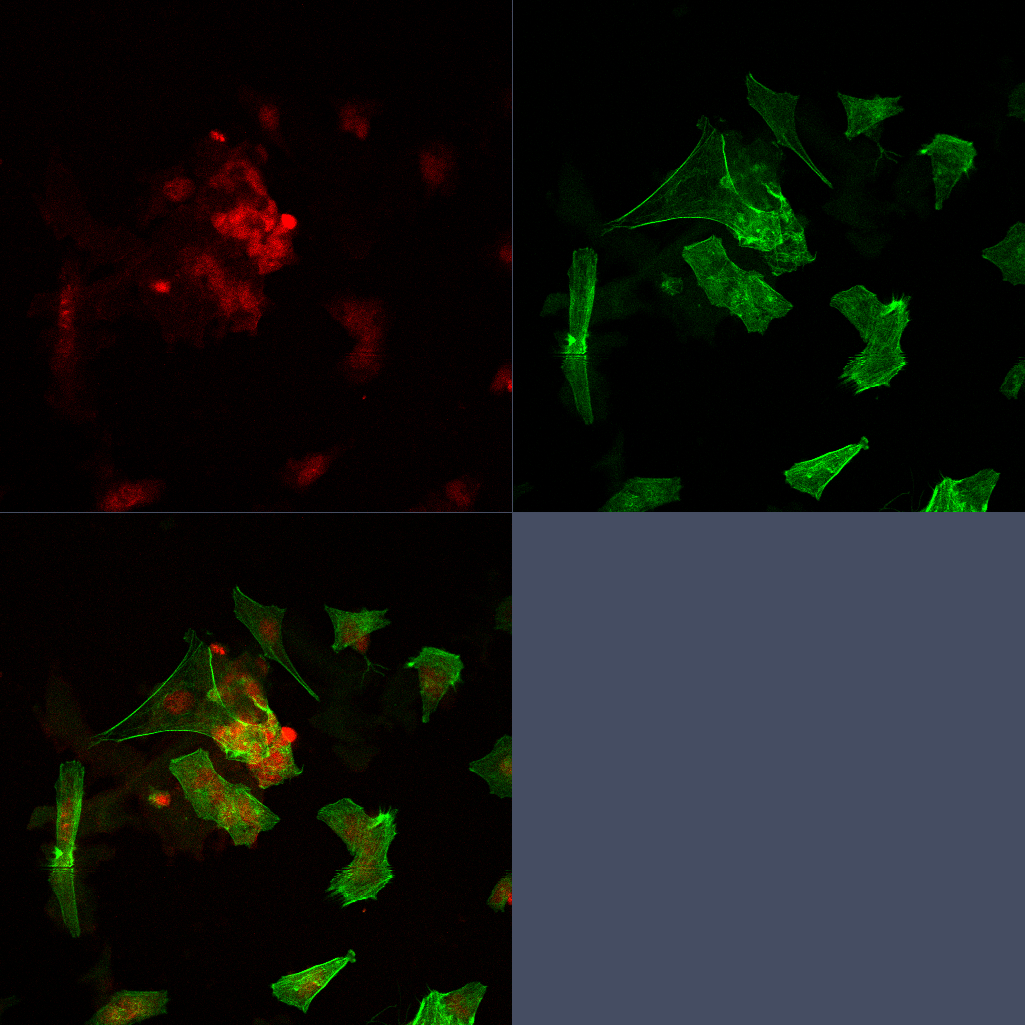

Supplement: Supplementary file 1 [file DataSheet1.ZIP › Figure 4/PrPs(106-126).jpg]

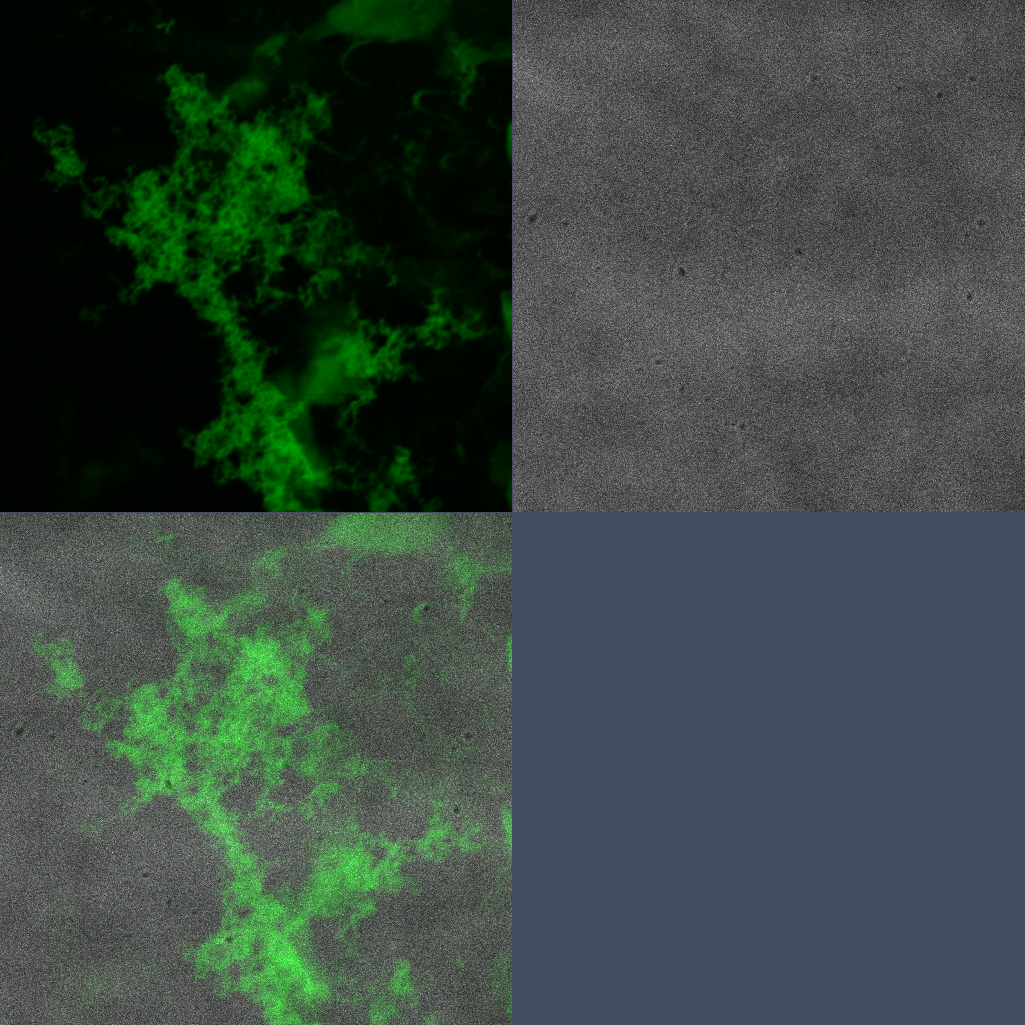

Supplement: Supplementary file 1 [file DataSheet1.ZIP › Figure 5/Fibrin polymer in presence of prion.jpg]

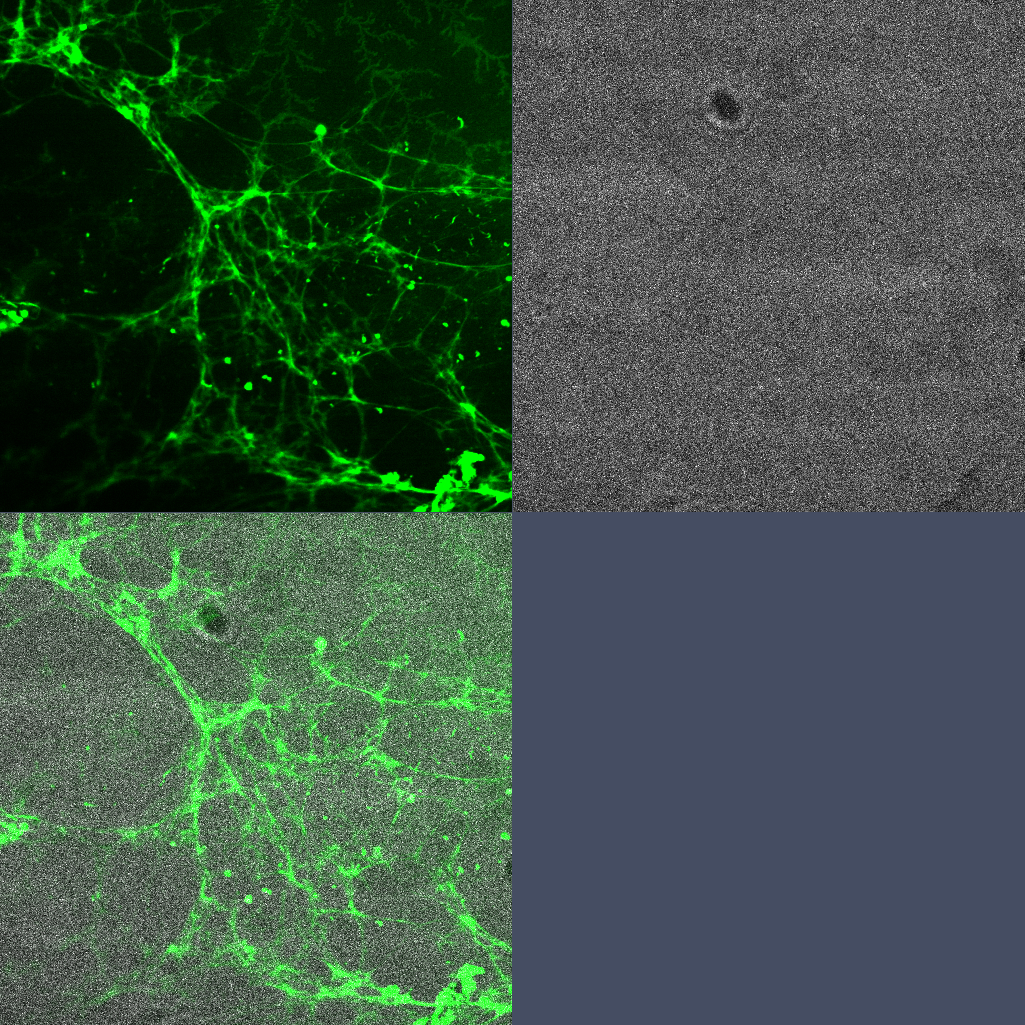

Supplement: Supplementary file 1 [file DataSheet1.ZIP › Figure 5/Fibrin polymer.jpg]

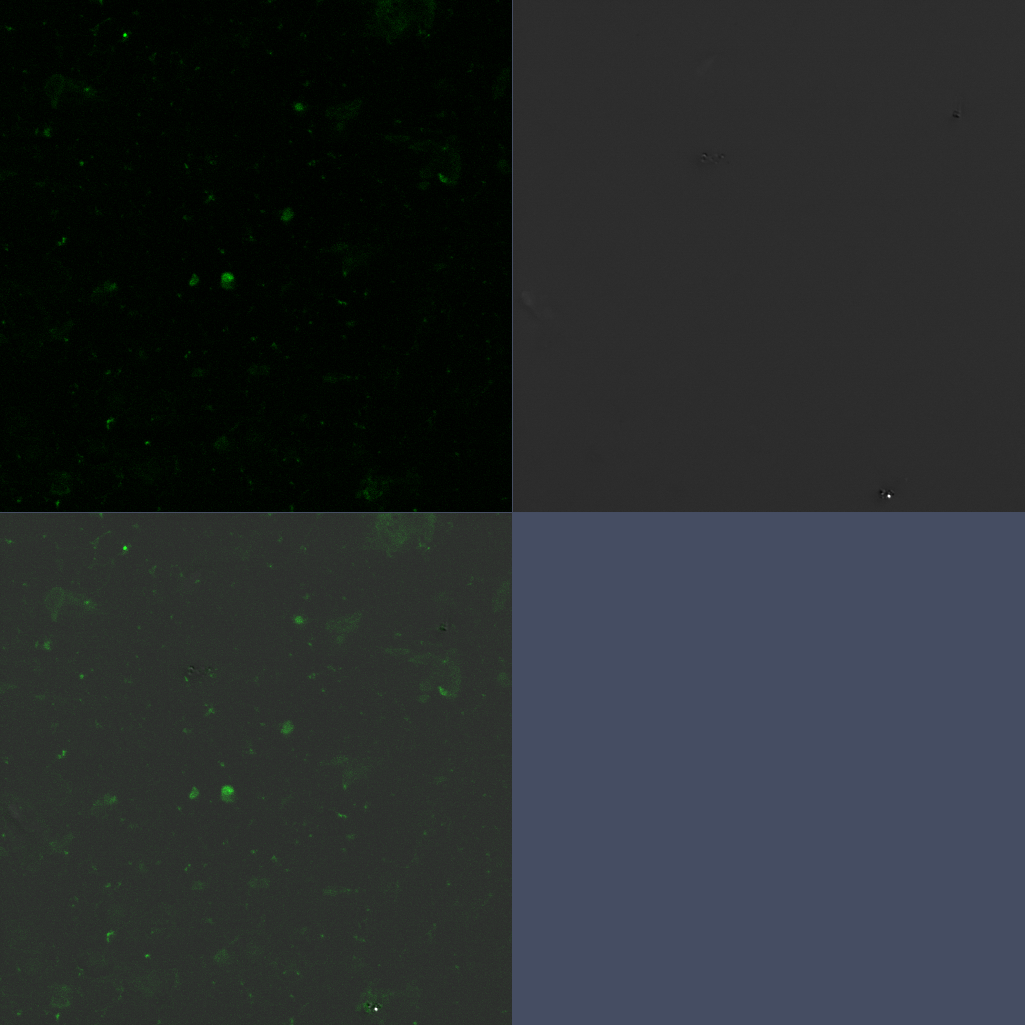

Supplement: Supplementary file 1 [file DataSheet1.ZIP › Figure 6/Fibrin polymer after lysis.png]

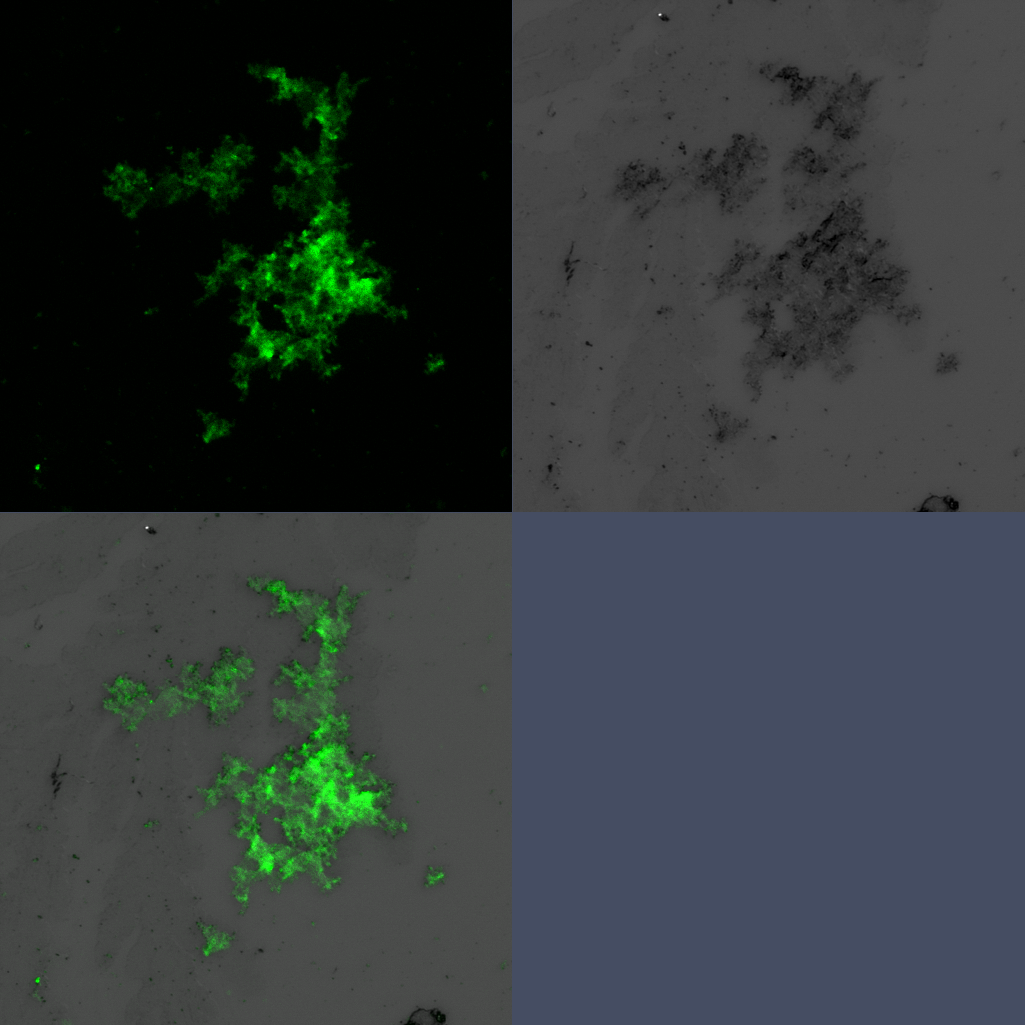

Supplement: Supplementary file 1 [file DataSheet1.ZIP › Figure 6/Fibrin polymer in presence of prion.png]

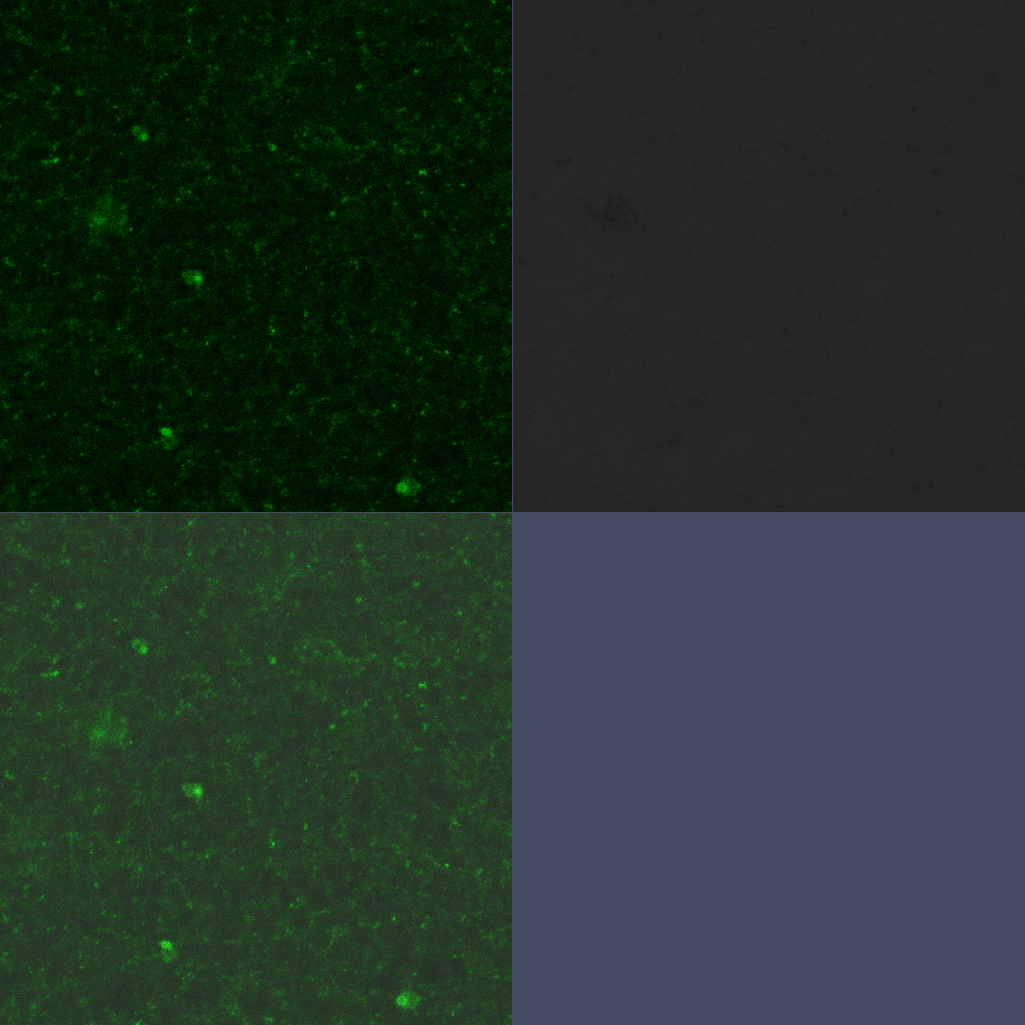

Supplement: Supplementary file 1 [file DataSheet1.ZIP › Figure 6/Fibrin polymer.png]

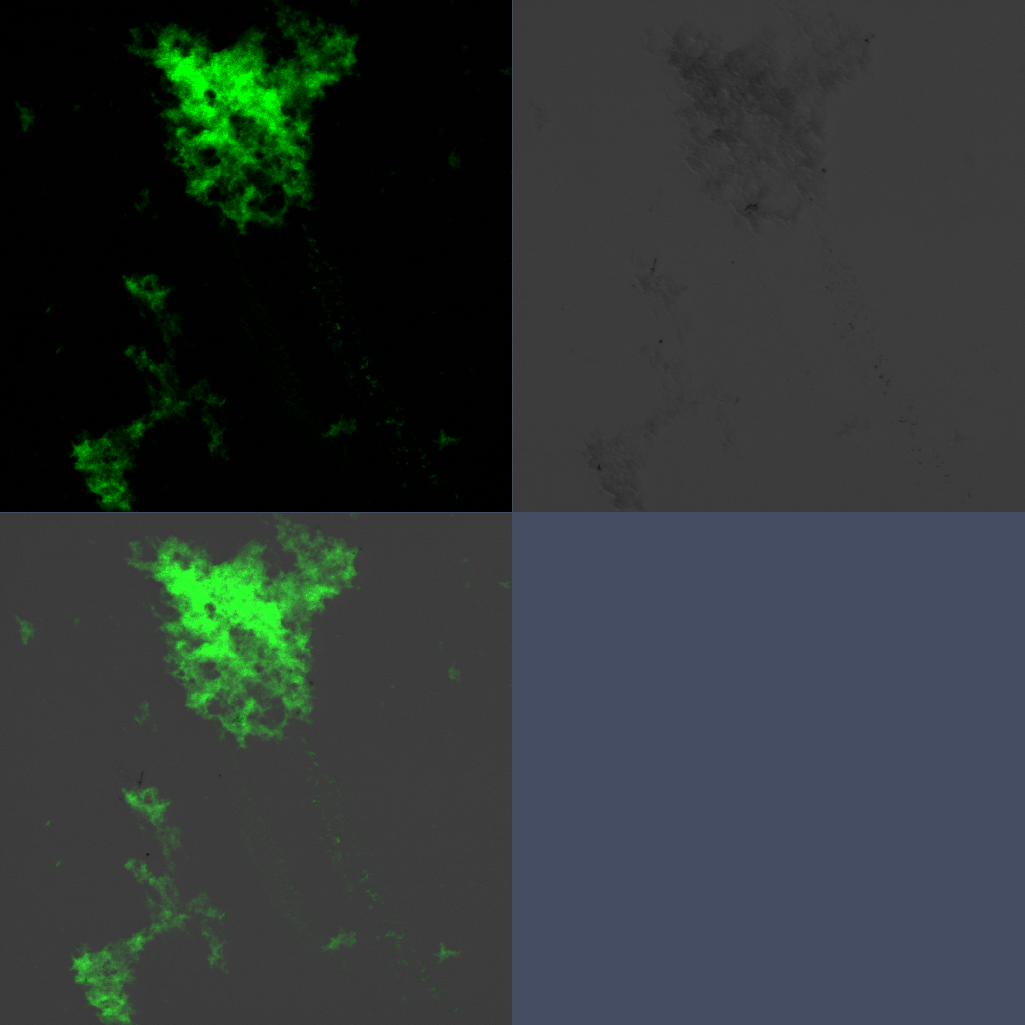

Supplement: Supplementary file 1 [file DataSheet1.ZIP › Figure 6/fibrinolysis of fibrin polymer made in presence of prion.png]
